# Design, Manufacturing, and Analysis of Periodic Three-Dimensional Cellular Materials for Energy Absorption Applications: A Critical Review

**DOI:** 10.3390/ma17102181

**Published:** 2024-05-07

**Authors:** Autumn R. Bernard, Mostafa S. A. ElSayed

**Affiliations:** Mechanical and Aerospace Engineering, Carleton University, Ottawa, ON K1S 5B6, Canada

**Keywords:** additive manufacturing, cellular materials, energy absorption, lattice topology, relative density

## Abstract

Cellular materials offer industries the ability to close gaps in the material selection design space with properties not otherwise achievable by bulk, monolithic counterparts. Their superior specific strength, stiffness, and energy absorption, as well as their multi-functionality, makes them desirable for a wide range of applications. The objective of this paper is to compile and present a review of the open literature focusing on the energy absorption of periodic three-dimensional cellular materials. The review begins with the methodical cataloging of qualitative and quantitative elements from 100 papers in the available literature and then provides readers with a thorough overview of the state of this research field, discussing areas such as parent material(s), manufacturing methods, cell topologies, cross-section shapes for truss topologies, analysis methods, loading types, and test strain rates. Based on these collected data, areas of great and limited research are identified and future avenues of interest are suggested for the continued maturation and growth of this field, such as the development of a consistent naming and classification system for topologies; the creation of test standards considering additive manufacturing processes; further investigation of non-uniform and non-cylindrical struts on the performance of truss lattices; and further investigation into the performance of lattice materials under the impact of non-flat surfaces and projectiles. Finally, the numerical energy absorption (by mass and by volume) data of 76 papers are presented across multiple property selection charts, highlighting various materials, manufacturing methods, and topology groups. While there are noticeable differences at certain densities, the graphs show that the categorical differences within those groups have large overlap in terms of energy absorption performance and can be referenced to identify areas for further investigation and to help in the preliminary design process by researchers and industry professionals alike.

## 1. Introduction

While the research field of *metamaterials*, defined as materials engineered with properties not easily found in nature, is vast and ever-growing [1,2,3,4,5,6], there is a subsection within this field of particular interest: cellular solids. Simply put, cellular solids are defined as “an assembly of cells with solid edges or faces, packed together so that they fill space” [7]. The cells themselves are usually an assembly of connected struts or plates and the tessellation to fill a volume can be stochastic or periodic, as is usually the case for foams and lattices, respectively [7,8,9,10,11]. Early work on cellular materials is thanks to Gibson and Ashby [7], whose book discussed the structure, properties (such as mechanical, thermal, and acoustic), and use of cellular materials, specifically foams and honeycombs. As is discussed in great detail in [9], micro-architected materials—of which cellular materials are a category—were developed to attempt to close gaps in the material property selection design space, which the manipulation of the chemistry and the microstructure of monolithic materials was not otherwise capable of. Specifically, for a given density, by manipulating the appropriate parameters, it may be possible to increase properties—such as stiffness, strength, or thermal conductivity—by a factor of 1000 or more [8].

These types of materials can offer a wide range of beneficial properties for many industries. For the biomedical industry, they can be employed within orthopedic implants for load-bearing or energy absorption applications or as scaffolding to optimize osseointegration [12,13,14,15,16,17,18,19]. Parisien et al. [20,21] investigated periodic cellular solids for their ability to promote better mature bone growth stimulation while Zhang et al. [22] found that a graded lattice structure with tapered struts increased permeability by over 27% as compared to the ungraded and untampered variation and bone density by over 50% as compared to the ungraded variation. Within the automotive industry, the appeal is for their use within structural components, such as crash mitigation, and to decrease noise conduction [23,24,25,26]. For the aerospace and defense industries, the appeal is similar to the automotive industry, in addition to the use within more advanced aircraft and propulsion systems [24,27,28,29,30,31]. Trudel et al. [30] achieved a 44% reduction in weight in a simple aircraft door hinge by utilizing lattice materials while Opgenoord and Willcox [32] applied their cellular structure design method to an aircraft bracket from the literature, achieving a 24% reduction in weight. Moreover, among other applications, lattice materials can be used in sports equipment as protective helmets, for thermal insulation and heat exchange, for electromagnetic shielding, for the protective packaging of delicate components, and as acoustic absorption barriers [24,33,34,35,36,37,38,39,40,41]. Chen et al. [42] presented a novel helmet design made with an auxetic lattice liner that was determined to reduce the head injury criterion (HIC) by over 72%, as compared to the allowable threshold HIC.

However, the issue of manufacturing arises with the desire for cellular materials with a high degree of controllability and repeatability; conventional methods for creating foams—such as the melt foaming method or investment casting—do not allow for sufficient control of mechanical properties, can be costly and inefficient, and can limit the achievable manufacturable geometrical complexities [43,44]. With the promise of the expansion of the material selection design space, the demand for such cellular materials—with highly controllable properties for a variety of applications—has pushed the search for new manufacturing techniques, particularly additive manufacturing (AM) [45]. As compared to the traditional techniques of manufacturing cellular materials, AM allows for the repeatable manufacture of these materials at a much smaller scale, with features on the order of micro- and nano-meters; a much higher complexity than subtractive or traditional manufacturing; and a more rapid build rate [44,46,47,48,49,50,51]. Zhao and Zong in [52] actually outline five *complexities* that AM has the potential of achieving: *shape complexity* (capable of building almost any geometry); *hierarchical complexity* (different design features can be included at multiple length scales); *functional complexity* (multiple parts can be manufactured assembled together); *material complexity* (multiple materials can be integrated into the design); and *metallurgical complexity* (metallic microstructures can be customized by adjusting heating and cooling regimes).

To refine the scope of this investigation, the following limitations were introduced during the review of the available literature:

- While *cellular materials* can be split into stochastic or periodic tessellation categories, the interest in this paper is for *periodic* cellular materials. The choice to eliminate stochastic variations from this work stems from the understanding that while their properties can be approximated, the recreation and exact prediction of properties are not possible due to the randomized nature of the cells [53]. Thus, periodic cellular materials are of greater interest for critical applications requiring a known response within a given environment.
- While tessellation and parameter variability of a lattice unit cell can vary in different Cartesian length scales, the focus of this paper is on 3D lattices versus 2D honeycombs or 1D counterparts.
- Our review includes closed or open-cell lattice topologies with sheets/plates or strut-like architecture, respectively, which includes but is not limited to, sheet-based (such as triply periodic minimal surfaces (TPMSs)) and periodic micro-truss architected lattices. In this work, the term *lattice*(*s*) is used to refer to all of these types of three-dimensional cellular materials, which each have a unique *unit cell* geometry that can be repeated in any and multiple of the principal Cartesian directions to form a *cluster*.
- There are also no limitations on the types of manufacturing technologies (traditional or additive) utilized for the fabrication of samples, so as to highlight the variety of techniques available; though, as previously briefly discussed, advances in AM technologies allow for the manufacture of cellular materials with higher geometrical complexities while offering the ability to reduce cost and increase reliability and repeatability.
- Finally, the interest is on *energy absorption* of 3D periodic lattices and, as such, the literature that did not adequately discuss the energy absorption of the investigated lattice(s) was not included in investigations and summaries of trends. For directly calculating energy absorption and associated parameters, the reader is directed to the other literature for the formulaic definitions [54,55].

This paper is organized as follows: after this introduction, Section 2 describes the method of collecting and organizing the existing literature; Section 3 discusses the types of materials and manufacturing methods utilized in the literature; Section 4 considers lattice topologies, including their classification system, the variation in number of topologies investigated per paper, and their design, including software types; Section 5 examines different characterization approaches, including experimental and numerical methods; Section 6 explores the strain rates and speed ranges—such as quasi-static or dynamic—utilized in the literature; and Section 7 explores energy absorption trends present in the published literature. The conclusion follows these sections.

## 2. Methods

**Figure 1:**
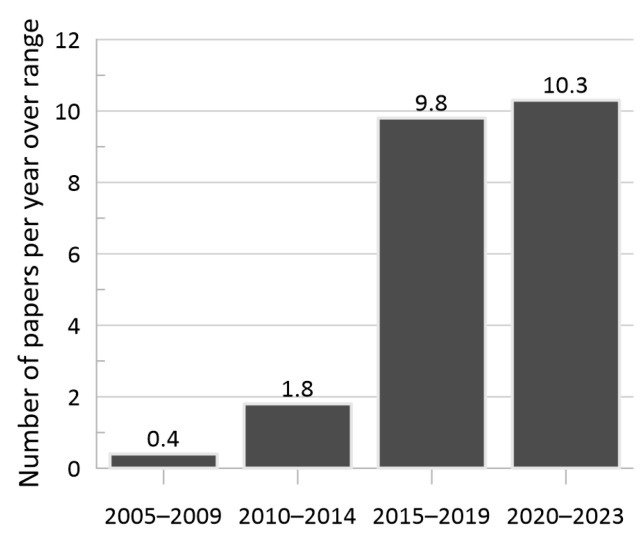
Average number of publications reviewed and summarized per year over specified time range.

With a focus on the characterization of energy absorption properties of periodic lattice materials, over 250 publications were reviewed—and then narrowed to 100 papers of interest based on the restrictions outlined in Section 1—spanning approximately the last two decades, where the average number of publications per year for the specified time range is provided in Figure 1. These publications were located using Google Scholar (Google, Mountain View, CA, USA) searches, using key search terms including “cellular materials”, “lattices”, and “energy absorption”, as well as reviewing reference sections of those papers and other review papers, such as [53,56]. Tools such as Connected Papers [57], Inciteful [58], and Litmaps [59] were also utilized to help discover the additional applicable literature based on the literature already identified as relevant.

Once a publication was identified as one of interest, all relevant data were collected including analysis approach of either analytical, computation, or experimental; commercial software package used for the analysis; impact test speed; and lattice-related information such as parent material, cell size, manufacturing method, among others. A summary of data collected for those papers of interest can be found in Table A1 at the end of the paper. Commentary regarding the results and trends illuminated by the information in that table is the focus of the following sections, and any challenges encountered during the data extraction process are discussed as they become applicable, notably in Section 4 and Section 7.

Overall, the data collected were reviewed using content and thematic analysis methods, as well as making inferences about the field based on the sample of numerical energy absorption data collected from 76 papers. It is noted that while there are emerging literature review and analysis methods using Artificial Intelligence (AI) and other automation techniques [60,61], only classical methods were adopted for data extraction and analysis purposes here.

It should also be noted that while there were limitations placed on the selection of papers of interest, papers that fell outside of the scope of what was defined in Section 1 are still referenced and discussed, particularly as a means of identifying additional gaps in research and looking for solutions from other disciplines.

## 3. Parent Materials and Manufacturing Processing Stages

This section discusses the importance of parent material in the design and manufacture of lattice materials, focusing not only on the general selection of the bulk material, but also on how the processing stage (i.e., manufacturing method) and post-processing stage (e.g., heat treatments) can influence the properties of this bulk material, thereby affecting the overall performance of the lattice material.

### 3.1. Pre-Processing Stage: Material Types

It is generally agreed upon that parent material is one of three dominating factors that influence the cellular material characteristics, meaning this choice is not insignificant in the scheme of cellular material examination [8]. Table 1 provides some information regarding potential motives for choosing one material over another.

**Figure 2:**
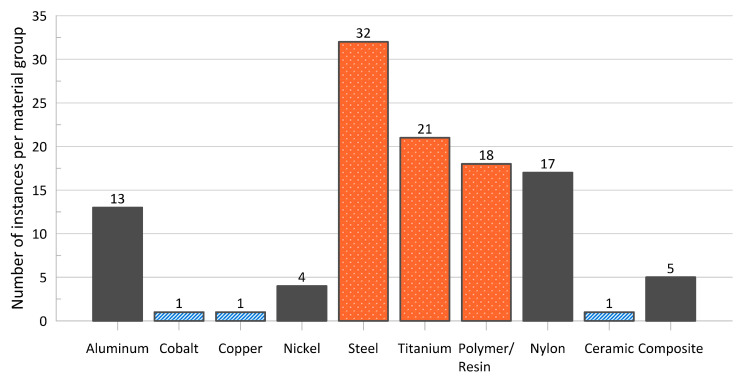
Distribution of material types utilized in the literature. Bars for maximum 3 values filled with orange speckle, bars for minimum value filled with blue diagonal lines, and all other bars filled in gray. Note that here, “Polymer/Resin” does not include nylon polymers since it is provided as a separate category, and the “Composite” category includes materials such as carbon fiber-reinforced and glass fiber-reinforced nylon materials.

As shown in Figure 2, steel and titanium alloy materials account for almost half of the research into the energy absorption characteristics of cellular materials while composites, nickel alloys, and copper alloy-based cellular materials are examined much less often. Given the maturity of the technologies available for manufacture and the availability of different materials, such a distribution is understandable. Steel and aluminum alloys are among the most widely used metals in the world [62,63,64], and availability of mature manufacturing techniques that are compatible with such materials (such as Selective Laser Melting) makes experimentation and analysis of lattice materials made from those parent materials more convenient [65,66]. The high use of nylon and other polymers/resins can also be explained by their low melting (and curing) temperature, general chemical stability, and good flow during manufacture following a polymer-suitable AM technique, such as Fused Filament Fabrication or Fused Deposition Modeling [67,68,69]. On the other hand, copper and its alloys are difficult to process via Selective Laser Melting as a result of the low laser absorption rate and high thermal conductivity or copper, meaning that research with this material has been relatively limited so far [44]. Additionally, there can be some difficulties associated with the additive manufacture of composite materials such as ensuring the even dispersion of discontinuous, chopped fibers (specifically, carbon nano-tubes) within the matrix of the composite [70,71]; void formation and poor fiber–matrix adhesion [72,73]; and extrusion-based techniques may experience nozzle clogging by the fibers [73]. While there has been research into continuous fiber-reinforced composites [74], the AM of continuous fiber composites comes with additional challenges [75]; all composites within the papers of interest consisted of discontinuous fibers or carbon nano-tubes.

**Table 1:** General description of properties of materials with some examples of applications.

Material Type	Descriptions, Advantages, and Disadvantages	General Applications
Steel and Steel Alloys [62,63,64]	Iron-based metals and alloys are the most widely used metal, particularly since they are relatively economical to produce. While in general they are versatile, they are also susceptible to corrosion. Low-Carbon Steel (LCS)—relatively low strength since increasing carbon content will increase strength; soft, good ductility; and least expensive of the carbon steels to produce. Medium-Carbon Steel (MCS)—high strength; high wear resistance; and high toughness. High-Carbon Steel (HCS)—hardest, strongest, and least ductile of the carbon steels; high wear resistance. Stainless Steel (SS)—good corrosion resistance; high strength and ductility.	LCS—nails; consumer goods; and cans. MCS—machine parts; rivets and other fasteners; and gears. HCS—cutting tools; files; and saws. SS—chemical and food processing (cutlery, kitchen equipment); petroleum industries; and health care (surgical equipment).
Titanium and Titanium Alloys [62,63,64,76]	Advantages: high strength-to-weight ratio; high elastic modulus; highly ductile; good corrosion resistance at high and low temperatures; high melting point; and low density as compared to iron. Disadvantages: expensive; chemically reactive with other elements at high temperatures.	Aircraft; engines; chemical and petrochemical industries; biomaterials (orthopedic implants); and dental applications.
Aluminum and Aluminum Alloys [62,63,64]	Advantages: high strength-to-weight ratio; low density; high ductility; good corrosion resistance; high thermal and electrical conductivity; non-toxic; non-magnetic; and abundant (it is the second most-used metal). Disadvantages: aluminum–lithium alloys, which are attractive for aerospace applications due to their high strength-to-weight ratios and excellent fatigue properties, are costly.	Containers; packaging; transportation industry (automotive, aircraft, aerospace, railroad); electrical products; and consumer appliances.
Copper and Copper Alloys [44,62,63]	Copper and its alloys are good for applications with multiple requirements, such as good electrical *and* mechanical properties. Advantages: good thermal and electrical conductivity; high corrosion resistance in multiple environments (e.g., seawater and industrial chemicals); and good wear resistance. Disadvantages: unalloyed, they are soft and ductile; low laser absorption rate and high thermal conductivity can make the AM of copper-based materials difficult.	Heat capacitor and heat exchanger applications.
Thermoplastics [62,63,70,77,78,79,80]	Generally cheap and abundant, thermoplastics are the default for use in Fused Deposition Modeling (FDM) AM technology. Acrylonitrile butadiene styrene (ABS)—high strength; high toughness; good abrasion, chemical, and heat resistance; and good electrical properties. Polyamide (PA, Nylon)—good mechanical properties; good toughness; good abrasion and chemical resistance; low coefficient of friction; and can absorb moisture/water (a limiting factor for design applications). Polyethylene (PE)—highest-volume polymer in the world. In general, good electrical and chemical properties; high toughness and ductility; low coefficient of friction; low moisture/water absorption; good ease of processing; low strength (can be a limiting factor for applications); and poor weather resistance. Low-Density PE (LDPE): high impact strength, toughness, and ductility. High-Density PE (HDPE): low cost, good availability, good ease of processing, and high performance-to-cost ratio. Ultra-High-Molecular-Weight PE (UHMWPE): good abrasion resistance; high toughness; and difficulty processing. Polylactide (PLA)—biodegradable; high strength; and low ductility. Polypropylene (PP)—good mechanical (including fatigue strength), electrical, and chemical properties; low weight; low cost; good availability; good ease of processing; high performance-to-cost ratio; resistant to heat distortion; and low moisture/water absorption.	ABS—automotive, aerospace, and medical device applications. PA, Nylon—gears; bearings; bushings; rollers; fasteners; zippers; electrical parts; tubing; guides; and surgical equipment. LDPE: packaging films (e.g., shrink film). HDPE: bottles (milk, juice); food containers; gas tanks; and garbage bags. UHMWPE: artificial knee and hip joints.
Composites [77]	Advantages of using such materials include weight reduction; high stiffness- and strength-to-weight ratios; tailorable properties (can align fibers in direction of load); redundant load paths (multiple fibers); can have increased/decreased thermal or electrical conductivity; and better fatigue life. Disadvantages of these materials include high material and fabrication costs; weak properties transverse to the fibers; matrix has low toughness and is subjected to environment, potentially leading to degradation due to those conditions; and difficult to analyze properties and non-destructive testing can be tedious.	

### 3.2. Processing Stage: Manufacturing Methods

The fabrication of cellular materials encounters unique challenges while ensuring geometric accuracy and overall efficiency, particularly at the micro- and nano-scales for periodic lattices. Rashed et al. [46] (and others [81,82]) outlined those manufacturing methods proven for the fabrication of micro-lattice materials, specifically metallic, including investment casting, deformation forming, woven and non-woven textiles, Selective Laser Melting (SLM), Electron Beam Melting (EBM), and a self-propagating photopolymer waveguide technique (using collimated ultraviolet (UV) light and a patterned mask). They mentioned that conventional methods of manufacture—for which they group investment casting, deformation forming, and woven and non-woven textile techniques—generally have a higher minimum requirement for relative density and cell size than those AM techniques discussed. Additionally, as mentioned in Section 1, AM allows for the fabrication of parts with much higher geometrical complexity, including those geometries developed from topology optimization [44,46,48,49,50,51,83]. Table 2 outlines additive manufacturing technologies per the seven categories outlined in the ISO/ASTM standard of [84] and provides some general advantages and disadvantages as well as suitable materials for those techniques.

**Figure 3:**
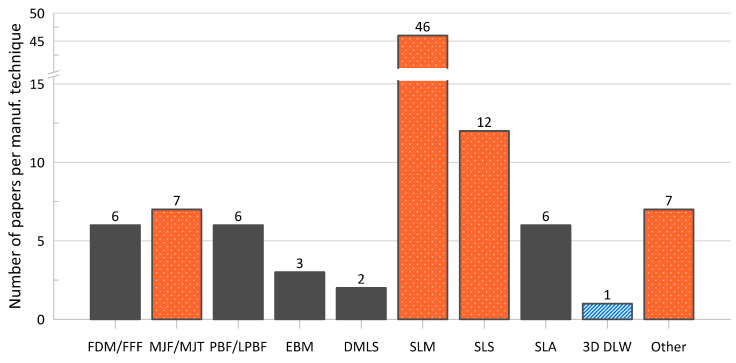
Distribution of manufacturing methods utilized in the literature. Bars for maximum 3 values filled with orange speckle, bars for minimum value filled with blue diagonal lines, and all other bars filled in gray. Note that here, “PBF/LPBF”, “EBM”, “DMLS”, “SLM”, and “SLS” are all listed as separate categories, where papers were classified based on the terminology they used and/or the type of 3D printer listed. The “Other” category includes collimated UV, water jet cutting, and those processes unspecified in the paper of interest.

Such a conclusion—that AM provides greater flexibility in the design and manufacture of cellular materials—is reflected in the distribution and variety of manufacturing methods encountered in the papers of interest, which is summarized in Figure 3. Additionally, though not generally a topic within the reviewed literature, there are advancements to AM technologies that allow for the fabrication of parts using multiple materials, opening the doors for this avenue of research within this field [85,86,87]. Indeed, the vast majority of methods identified follow AM techniques and other, more conventional, methods are limited; one of the conventional methods utilized was water jet cutting of titanium alloy plates by Dong et al. [88] to fabricate their lattice samples, snap-fitting and vacuum brazing the joints. 

As the influence of parent material on the performance of a lattice material is non-negligible, it is important to note that the parameters associated with the AM of specimens (e.g., laser power, laser exposure, layer thickness) can affect the final microstructure of the parent material. While the influence of those parameters during AM is not limited to only lattice materials, the unique geometry of these materials means that investigations with AM cellular materials should not be disregarded. In [89], Tsopanos et al. investigated the effect of laser power and laser exposure time during the Selective Laser Melting manufacture of body-centered cubic (BCC) lattices on their mechanical performance. They noted that low laser powers reduced the strength of struts while higher laser powers yielded struts that performed comparably to the bulk material properties. Sallica-Leva et al. [90] manufactured titanium alloy lattice specimens using Selective Laser Melting and investigated the effects of low- and high-energy inputs on their microstructure and mechanical performance. They found that the samples manufactured at higher input energy had higher mechanical properties, as compared to the low-energy samples at the same relative density. They used the increase in oxygen and nitrogen in the struts of those high-energy samples as rationale for the increase in mechanical properties.

**Table 2:** Additive manufacturing technologies per the seven categories outlined by ISO/ASTM 52900 [84], including advantages, disadvantages, and types of materials suitable for the technique. Adapted from [69,76,79,80,81,91,92,93].

ASTM Categ. ^1^	Technique	Advantages	Disadvantages	Materials	Build Volume Size (X mm × Y mm × Z mm) Resolution: (μm)
BJT ^2^	3D inkjet	Free of support/powder bed acts as integrated support structure Design freedom Large build volume High print speed Relatively low cost Large range of material options	Fragile parts with limited mechanical properties May require post-processing Rough or grainy appearance	Polymers Ceramics Composites Metals Hybrid	Vol: Small to large (<4000, <2000, <1000) –
DED	LD	Reduced manufacturing time/cost, high material deposition rate, and high material utilization Accurate composition control Highly controlled grain structure/microstructure High-quality parts Excellent mechanical properties Excellent for repair and retrofitting applications	Surface quality and speed requires fine-tuning/balance Limited to metals/metal-based hybrids Limitations with regards to complex shape with fine details	Metals Hybrid	Vol: Small to large (600–3000, 500–35,000, 350–5000) Res: (250)
	LENS				
	EB				
	PAM				
MEX	FDM/FFF/FLM	Inexpensive, widespread use Scalable High speed Simplicity Can build fully functional parts	Long build time Vertical anisotropy, weak mechanical properties Step-structured surfaces (due to layer-by-layer build) Not amenable to fine details Limited materials	Polymers Composites	Vol: Small to medium (<900, <600, <900) Res: (50–200)
MJT	3D inkjet	High accuracy of droplet deposition No/low material waste Multi-material, multi-color possible	Support material is often required and cannot be recycled/reused Mainly photopolymers and thermoset resins can be used Post-processing could damage thin or small features	Polymers Ceramics Composites Hybrid Biologicals	Vol: Small (<300, <200, <200) –
	DIW				
PBF	EBM	Fine resolution, high quality, accuracy Small footprint Powder bed acts as integrated support structure Large range of material options Polymer and metal powder can be recycled	Relatively slow build rate Lack of structural integrity, rough surface finish Size limitations Expensive machines Finish depends on precursor powder size	Polymers Ceramics Composites Metals Hybrid	Vol: Small (200–300, 200–300, 200–350) SLS/SLM Res: (80–250)
	DMLS				
	SLS/SLM				
SHL	LOM	High fabrication speed Low cost Ease of material handling Reduced tooling and manufacturing times Excellent for manufacturing large structures Multi-material, multi-color possible	Strength and integrity of parts depend on adhesive used Finishes may require post-processing Inferior surface quality and dimensional accuracy, warpage possible Limited material use, limitations in design complexity High material waste	Polymers Ceramics Metals Hybrids	Vol: Small (150–250, 200, 100–150) LOM Res: varies based on sheet thickness
	UC/UAM				
VPP	SLA	High fabrication speed Excellent accuracy, fine resolution Excellent surface finish and details, high quality	Low shelf-life Poor mechanical properties, post-curing required to enhance strength Expensive Slow build process Requires supports and post-processing to remove them	Polymers Ceramics	Vol: Medium (<2100, <700, <800) SLA Res: (10)
	DLP				

^1^ Liquid, powder, and solid bulk material. ^2^ Liquid- and powder-based. Acronyms in table: Vol.—Volume; Res.—Resolution; BJT—binder jetting; DED—direct energy deposition; MEX—material extrusion; MJT—material jetting; PBF—powder bed fusion; SHL—sheet lamination; VPP—vat photopolymerization; LD—laser deposition; LENS—Laser Engineered Net Shaping; EB—Electron Beam; PAM—Plasma Arc Melting; FDM—Fused Deposition Modeling; FFF—Fused Filament Fabrication; FLM—Fused Layer Modeling; DIW—Direct Ink Writing; EBM—Electron Beam Melting; DMLS—Direct Metal Laser Sintering; SLS—Selective Laser Sintering; SLM—Selective Laser Melting; LOM—Laminated Object Manufacturing; UC/UAM—Ultrasound Consolidation/Ultrasound Additive Manufacturing; SLA—Stereolithography; and DLP—Digital Light Processing.

Even the geometry can have an effect on the microstructure development during AM. In [94], del Guercio et al. evaluated the mechanical performance of titanium alloy lattice structures and also performed a microstructural analysis of the specimens. They noted that “the rapid and directional solidification phenomena during the AM process can define the local microstructure”, noting differences between the microstructure of three topologies (dode thin, G-structure, and rhombic dodecahedron) at similar strut diameters, but finding similarities when the strut diameters varied from one topology to another. They explain this peculiar result as perhaps related to the thermal history during AM. Pyka et al. [45,95] noted differences in surface roughness between the top and bottom surfaces of non-vertical struts and explained in [45] that the heat flow and staircase effect play a role in such a phenomenon; the bottom surfaces of struts see a greater accumulation of powder particles during AM due to variations in heat flow while the surfaces of vertical struts are more uniform. Dong et al. [96] also discussed this phenomenon. In [97], Liu et al. discussed variations in material microstructure between the struts and nodes of a BCC-type lattice, even noting differences in microstructure based on the location within those bodies (e.g., top or bottom of node or angled strut). They rationalized this *gradient microstructure* as arising from “varied cooling rates resulting rom the lower thermal conducting of the un-melted powder particles in contact with built regions”.

### 3.3. Post-Processing Stage: Treatments

While it appears that the majority of papers performed no post-processing treatments on the as-built lattices prior to experiments, some did note investigating the effect of post-processing treatments [49,98,99,100]; others simply utilized post-processing treatments to reduce residual stress in the as-printed part before performing experiments [101,102]. In 2016, Maskery et al. [49] investigated the mechanical and energy absorption characteristics of uniform and functionally graded BCC lattices. They performed heat treatments on the as-built SLM aluminum samples and found that the post-manufacturing process allowed the lattices to exhibit a more ideal stress–strain behavior of cellular materials as compared to the as-built lattices. In 2017, Maskery et al. [98] manufactured “double gyroid” TPMS Al-Si10-Mg lattices and investigated their compressive deformation response, including their energy absorption capabilities. They also performed a post-manufacturing heat treatment, noting that it eliminated the brittle fracture and its low-strain failure. They concluded by suggesting that a smaller cell size should be chosen so as to avoid low-strain failure due to localized fracture and crack propagation but noted that the resolution of the additive manufacturing technique must also be considered to ensure the manufacturability of the lattice. They also compared to previous work with BCC lattices [49], mentioning the heat-treated double gyroid lattices absorbed almost three times as much specific energy by a 50% compressive strain as those BCC lattices. Köhnen et al. [99] experimentally investigated the tensile, compressive, and fatigue responses of lattices made of either face-centered cubic with vertical struts (FCC-Z) or hollow spherical unit cells, having also prepared annealed samples of the FCC-Z topology to investigate the effects of that heat treatment. They found that the annealing had no significant benefit to the tensile or fatigue properties of the FCC-Z SLM lattice: it decreased the maximum force and elongation to fracture, and the slope of the force number of cycles line during fatigue investigations was steeper for the annealed samples (thus, for a given force, the annealed samples would fail in fewer cycles). Jin et al. [100] manufactured BCC and octet Ti6Al4V lattices by SLM and performed a variety of heat treatments (750 °C–1050 °C and hot isostatic pressing) to investigate their effect on resulting mechanical properties. They noted that for the bending-dominated BCC, a higher heat treatment temperature (920 °C) gave better properties, while a lower temperature (750 °C) was better for the stretching-dominated octet.

The investigation into the effects of treatments to the lattice in the post-processing stage appears to be well underway for the general microstructural and basic mechanical characterization of additively manufactured lattice materials but is still lacking when it comes to specific influences on the energy absorption of lattice materials [45,90,95,103,104,105,106]. Some investigations concerning, specifically, additively manufactured lattice materials (outside of the interest in energy absorption capabilities) include Wauthle et al. [106], who investigated the effects of build orientation and heat treatment on both the microstructure and mechanical properties of SLM Ti6Al4V lattice samples. Having looked at five orientations and three heat treatments, they noted the importance of avoiding horizontal struts in relation to the build direction and that a hot isostatic pressing heat treatment could be beneficially used for static and dynamic loading situations. De Formanoir et al. [105] investigated the effects of chemical etching on the microstructure and mechanical properties of EBM Ti6Al4V lattice structures, finding that the parts treated with the chemical etchant had reduced surface roughness and an increase in relative stiffness as compared to as-built parts. On the chemical etching note, Pyka et al. [45] investigated the effect of chemical etchant on an SLM Ti6Al4V strut-based lattice material. They found that the concentration of the etchant was most influential on surface roughness reduction and that the strut thickness could decrease the effectiveness of the treatment.

## 4. Lattice Material Topologies

### 4.1. Topology Classification

Bhate in [107] ponders four key questions regarding cellular material design, the first of which is *what is the optimum unit cell*? Such a question appears straightforward enough but quickly becomes very difficult given that there is technically a nearly infinite list of unit cell topologies to choose from, each providing their own unique set of characteristics. Helou and Kara in [53] mentioned that there tends to be shortcomings in definitions of lattice structures, which either ignore stochastic possibilities or non-strut-based configurations. Indeed, with a nearly infinite list of topologies to choose from, it becomes difficult to come up with a consistent naming convention for new designs. Attempts have been made, such as in [108], which proposed a framework for describing trusses using a hierarchical language system with a lexicon, grammar, and syntax, having derived it to describe the position of the nodes of a lattice in space and their connectivity (struts). Additionally, a three-tiered classification system was proposed in [109] based on *tessellation* (division of the design volume into smaller segments); *elements* (use of beams and/or shells or plates within the smaller volume segment); and *connectivity* (how the elements are connected within the smaller volume segment).

**Figure 4:**
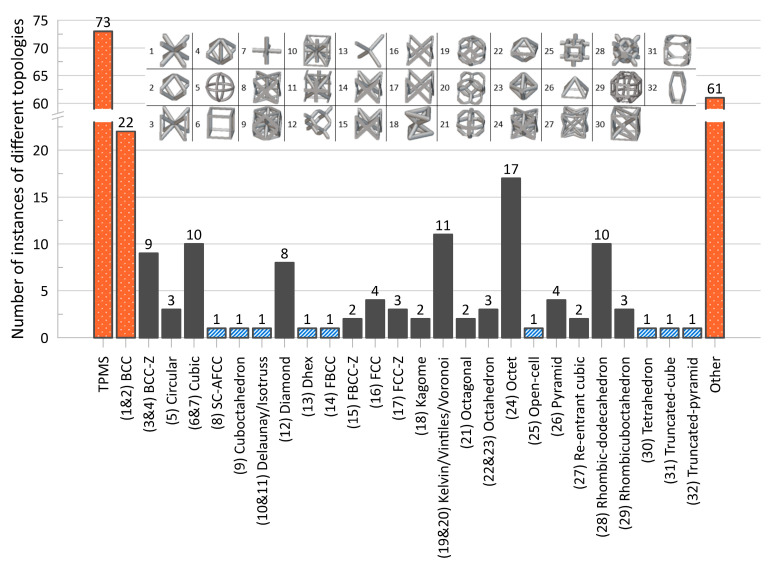
Distribution of topologies investigated in the literature. Bars for maximum 3 values filled with orange speckle, bars for minimum value filled with blue diagonal lines, and all other bars filled in gray. “TPMS” has a more detailed breakdown in a separate figure; “Circular” also includes variations in the strut-based circular topology (e.g., semi-circle, cross semi-circle); “Diamond” includes “Dfcc”; and “Other” includes topology-optimized geometries as well as unique geometries created for the purposes of the paper of interest (e.g., “Twisted-octet”, “CLL”, “cross-chiral honeycomb”) and plate-/shell-based topologies (e.g., “BCC-6H”, “BCC-12H”) are also grouped within this category. Numbers in brackets preceding topology names are associated with the respective numbered topology unit cell view. *nTopology* (nTop, New York City, NY, USA) [178] was utilized to create the majority of the illustrations of the strut-based cells; *Ansys SpaceClaim* (Ansys Inc., Canonsburg, PA, USA) [179] was utilized for the circular and rhombicuboctahedron cells.

**Figure 5:**
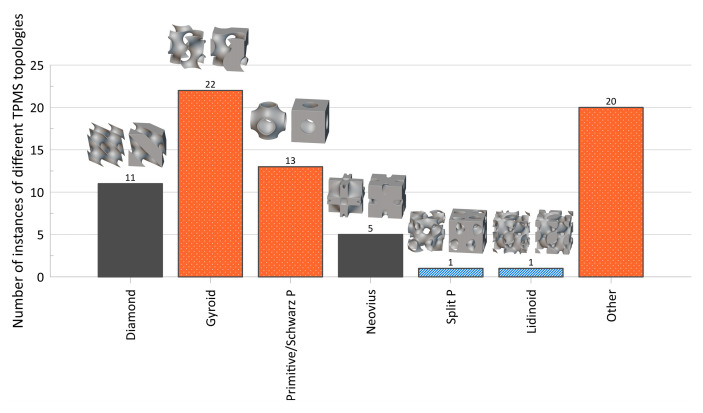
Distribution of TPMS topologies investigated in the literature. Bars for maximum 3 values filled with orange speckle, bars for minimum value filled with blue diagonal lines, and all other bars filled in gray. Here, the “Other” category includes TPMS-like sheet topologies, TPMS-BCC, “FRD”, and “IWP”. Images of TPMS topologies are provided above the respective bar value and include illustrations for (left) sheet-based/matrix phase and (right) a version of the skeletal-based/network phase. *nTopology* (nTop, New York City, NY, USA) [178] was utilized to create the illustrations of the TPMS topologies.

And yet, the obstacle of inconsistent naming conventions for topologies was encountered while compiling publications and their associated data. For the most part, a standard name was used across the literature for a given topology, but, still, sometimes the label provided for an illustrated topology did not match the convention other publications had been following. In an attempt to be as clear as possible on the distribution of topologies addressed in the literature, in publications where the label and topology photo did not match what appeared to be the standard in other papers, the label was switched to reflect the topology being analyzed more accurately. Some examples in the literature to highlight these naming inconsistencies are outlined in the following list, where it should be noted that the examples pulled from the literature are not meant to be a critique of the choice of terminology by those authors, only a critique of the general inconsistencies, which can make research and comparison more difficult. Figure 4 and Figure 5 provide some visual representations of these topologies (as noted).

- The use of the terms “sheet-” or “skeletal-based” for TPMS topologies is not universal. For example, in [98] (who also distinguishes between sheet and skeletal using matrix phase and network phase), what may have been identified as a skeletal gyroid and sheet gyroid by other sources [110,111,112,113,114,115] are termed a *gyroid* and *double gyroid*, respectively. Some additional terms in the literature for “skeletal” in such a context include “solid” [116]; “ligament” [117]; and “primary” and “secondary” [118]. For differences between sheet and skeletal TPMS topologies, see the illustrations in Figure 5.
- In [119], the topologies BCC and BCC-Z (such as is identified in [120,121,122,123]) are termed *octahedral* (or [±45°]) and *pillar-octahedral* (or [0°,±45°]), respectively. While these terms are used for the same geometry (see Figure 4(1) and Figure 4(3), respectively), these terms may also be used to describe geometries with slight variations (see Figure 4(2) and Figure 4(4), respectively), such as in [124,125,126]. This has also been noted by Noronha et al. [127] during their comprehensive review of hollow-walled lattice materials.
- Tancogne-Dejean et al. [128], Jin et al. [100,129], and Alberdi et al. [130] identify an octet topology (Figure 4(24), as in [131,132,133]) as FCC. In naming the combinations of multiple elementary strut-based unit cells, they [128] utilize the elementary names (simple cubic—SC, BCC, FCC) and a certain combination, termed as *SC2-BCC*, the *Delaunay* or *isotruss* geometry is created (Figure 4(10)), without reference to those particular names that are used elsewhere in the literature [134,135,136,137]. The use of a name that indicates the elementary cell combination is used in the other pieces of literature, such as [138], but the method of creating such a term—such as the abbreviations used—is not always consistent.
- Other variations in naming combinations of pre-existing unit cells include the term F2FCC-Z [139] to describe the FCC-Z cell [120,121] (Figure 4(3)); the term SC-BCC [128] to describe what might be called *star* or *cubic center* in other sources [20,140,141]; and the term F2BCC [142], which may be described as Face- and Body-Centered Cubic (FBCC) in the other pieces of literature [20] (Figure 4(14)).
- There also tends to be some variation in the identification of the tetrakaidecahedron topology (Figure 4(19)). Some pieces of the literature may reference it as a tetrakaidecahedron, while others may use the term *Kelvin*, sometimes referencing the other term and other times making no mention of it [50,143,144,145,146,147,148,149]. The terms *Voronoi* or *truncated octahedron* are also used to describe a geometry that is identical to tetrakaidecahedron/Kelvin [134,150,151,152]. By shifting the unit cell, an apparently new geometric configuration is created (Figure 4(20)), generally termed *Vintile*(*s*) [20,140].
- As a final note, such inconsistencies are not limited to the written literature but also spread into design software for lattices. For example, the Rhinoceros/Grasshopper plugin *IntraLattice* [153] uses the terms *grid*, *X*, *star*, and *cross* for topologies otherwise identified as *cube/cubic*, *BCC*, *SC-BCC* or *cubic center*, and All Face-Centered Cubic (*AFCC*) (Figure 4(6) and Figure 4(1) for cubic and BCC topologies, respectively).

The distribution of the number of instances a particular topology was examined in the literature is provided in Figure 4, with a more detailed breakdown of TPMS topologies provided in Figure 5. As mentioned, it should be noted that the name utilized in a given paper may not match how it was categorized in the figure as an attempt was made to keep naming consistent with how the majority of papers presented its geometry and geometries that deviated had names changed to a term more consistent across the literature.

One takeaway from Figure 4 is that there are many topologies classified within the “Other” category. This label was used to group not only plate-based topologies (of which there were a few, including [70,154,155,156,157]) but also newly designed unit cells [158], topology-optimized designs [43,159,160], and bio-inspired topologies [161], which may have only been examined just once, within the paper of interest. With the advent of new topologies, particularly as new topologies emerge from the increasing use of multi-objective and topology optimization, and of bio-inspired topologies, it can be seen from Figure 4 that while some “conventional” topologies (e.g., *octet*, *cubic*, *BCC*) are prominent in the literature, new topologies (in the “Other” category) are also increasingly of interest.

On this note, Helou and Kara [53] critiqued the lack of a definition for *new* versus *existing* topologies, as many of the existing topologies are simply slight variations of other existing topologies (such as the Delaunay or isotruss topology being a combination of two cubic cells and a BCC cell, as in [128]), and that this leads to the need for classifications on how to distinguish between what is indeed a new topology and what is simply a variation of existing topologies. This classification also links to what appears to be a general lack of naming system for topologies; as new topologies get designed, how is consistency and clarity ensured in the naming of those topologies, such that the literature focusing on those topologies can be easily located? As highlighted with some examples previously, if there are inconsistencies in the identification of a particular topology, finding the literature concerning that particular geometry requires knowing a variety of terms. Some considerations when adapting or developing a naming and classification system may include the following, which considers previous classification systems presented in [108,109]:

- Shape:
  - *Elements*: Beams, plates, or surfaces or based on a mathematical equation (e.g., TPMS).
  - *Topology optimization:* Is it possible to classify unique topologies arising from topology optimization processes [162,163,164,165]? Yang et al. [159] utilized topology optimization to create a unique unit cell, which they recognized had a similar microstructure to that of a cuttlefish bone, calling the cell “CLL” (cuttlebone-like lattice).
  - *Biomimetic structures:* How does the classification system handle unique topologies arising from biomimetic approaches [166,167,168,169]? Bhat et al. [170] began their *nested* lattice creation with a sea-urchin-inspired shell lattice, noting that it is nearly a replica of the Schwarz-P TPMS topology.
- Multi-scale and multi-morphology:
  - *Hierarchy*: Is it possible to specify a hierarchical component(s) of the cellular material in a clear manner? Lv et al. [171] investigate the mechanical properties of hierarchical lattices, from zeroth-order to second-order.
  - *Heterogeneity*: Is it possible to clarify the presence of multiple separate topologies within one cluster or part, as in Alberdi et al. [130] and Yu et al. [172], who both created clusters of octet cells with patterns of either BCC or rhombic dodecahedron unit cells, respectively, spread throughout. Or in Bhat et al. [170], who *nested* truss-based topologies (BCC, octet, rhombic dodecahedron) within the Schwarz-P TPMS topology. Bernard et al. [173,174] created unique lattice clusters by layering different topologies, terming these configurations both “sandwich lattices” and “multi-layer multi-topology (MLMT)” lattices.
- *Tessellation*: Periodic or stochastic; uniform or functionally graded.
  - *Cell symmetry, cell envelope*: Can the system account for both cell symmetry and cell envelope shape (i.e., variations in 2D polygons or 3D polyhedral unit cell envelopes)?
- *Multi-material*: As in [52] by Zhao and Zong, Pan et al. [175] predict the development direction of lattice structures to include, among others, designs with multiple materials. Thus, can the system accommodate the naming of topologies consisting of multiple materials (particularly two or more solid-phase materials, which Pan et al. cite as possible due to the layer-by-layer additive manufacturing technology)? Indeed, such dual- or multi-material investigations have begun. In [176], Li et al. fabricate 4130 steel lattices by SLM, where the remaining volume is filled with epoxy. Mueller and Shea in [177] take a different approach where the beams of the lattice are fabricated with two materials: a brittle core and flexible exterior.

Such a classification and naming system could be presented as an invaluable resource to the cellular material community as a web page of an interactive library of geometries, organized in multiple ways, allowing for ease of identification of the topology of interest.

### 4.2. Quantity of Topologies per Paper

**Figure 6:**
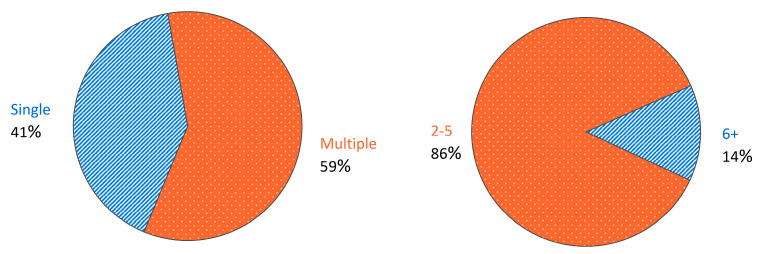
(**Left**) Distribution of the literature investigating multiple different topologies in one publication; (**Right**) distribution of number of topologies investigated when investigating more than one topology.

Figure 6 provides a summary of the distribution of the literature examining only one topology versus multiple topologies as well as, if it does examine multiple topologies, how many topologies it does end up examining. While over half of the literature investigated multiple topologies, a large majority of them investigated five or fewer topologies. Averaging the number of topologies across all papers yields an average of 2.5 topologies per paper while averaging the number of topologies across only those papers that investigate multiple yields 3.6. Since there can be many experimental or numerical model differences between one paper and another, which can lead to difficulties in comparing the results and conclusions drawn, investigating multiple topologies at once would be ideal. Understandably, limitations including cost and time can make investigating multiple topologies difficult. As will be discussed in a subsection of Section 4.3, the availability of design software for lattice geometry may also be prohibitive to the investigation of many topologies.

### 4.3. Topology Designs

This subsection is divided into four parts, the first of which focuses on software available for lattice design. The second part discusses the mathematical design of TPMS structures while the third focuses on struts, a structural design element of truss lattices. Finally, a discussion of lattice clusters and the application of lattices to real-world components is presented in the final part.

#### 4.3.1. General Lattice Design Software

Prior to any sort of characterization or examination of cellular materials, the geometry itself must be generated, whether for use in finite element analysis or for manufacturing and experimental testing. It is possible to generate the geometry (1) explicitly by using traditional computer-aided design (CAD) software or (2) implicitly by using mathematical functions (particularly for TPMS structures) [180,181]. Yet, given the apparent importance of such a step in the investigation of these materials, it is quite difficult to determine the most efficient method of geometry generation or a method that provides flexibility for a large variety of geometries. Indeed, even with great advancements in AM technology, allowing for the manufacture of materials as geometrically complex as cellular materials, it is still difficult—and sometimes impossible—to find a software solution capable of designing them [180,181,182,183]. Curiously then, it was noted during the review that many authors do not even provide this information. Of those papers that do provide such information, the methods provided in Table 3 were some of those identified as having been used for geometry generation.

While Table 3 is by no means an exhaustive list, note that it also includes methods identified in papers outside of the scope of *3D periodic cellular materials for energy absorption* such that it could be useful for the reader to be aware of the methods currently available. For information on methods specific to functionally graded lattice structures, the reader is directed toward [221], which provides a table of additional tools for such lattices. A review of design tools for AM, which also provides ease-of-use and cost for those CAD tools, (though not specifically for lattice materials) is provided in [222].

For some of the literature performing both experiments and numerical investigations, after manufacturing samples for experiments, they utilized the geometry of the as-built sample to create the model for the finite element analysis [124,223]. This allows for a more accurate representation of the as-built cellular material, which has been cited as one of the possible reasons for differences in experiments and finite element analysis [224]. Indeed, to reduce the differences between as-designed and as-built structures, other methods have also been used. Smith et al. [125] modeled the struts with multiple collinear beams of different diameters (beams nearest to the nodes had larger diameters) to more accurately “account for the lack of contact between the struts around the nodal region within a unit cell”. Outside of energy absorption investigations, Park et al. [150] ran multiple Finite Element Analyses (FEAs) to determine the optimal array type, relative density, and unit cell type before then performing experiments on samples using SLM. Noting that initial fracture in the specimens started at locations of high stress concentration, they added fillets into the design of the transition between nodes and struts of the lattices. They saw a reduction in stress concentration as well as an increase in compressive strength. Nazir et al. [225] investigated the effect of fillets on the performance of Kelvin unit cells by filleting an increasing number of connections between struts. They noted a 20% improvement in energy absorption from the un-filleted Kelvin cell to the fully filleted Kelvin cell, seeing also that the failure location shifted from the sharp edges at the joints to other locations with the addition of fillets. Some of these design approaches within FEA models are also referenced in Section 5.3.

#### 4.3.2. Triply Periodic Minimal Surface (TPMS) Lattice Design

As mentioned, TPMS lattices are generated implicitly using mathematical equations; general forms of these equations for TPMS lattices, as in Figure 5, have been highlighted in Table 4, where additional variations in the equations, as seen in the investigated literature, have also been provided. Note that these equations capture the general form used in the literature (e.g., sines and cosines) and do not necessarily reflect the exact form presented in the reference source, particularly in relation to the level-set values utilized, since they were put in the form f(x,y,z)−C(x,y,z)=0, which is required for MSLattice [206].

Described simply, a zero-level set surface (“iso-surface”) is defined when f(x,y,z)=0 and divides a given volume into two separate volumes of f>0 and f<0 [118,226,227]. Sheet-based TPMS can be created by thickening the iso-surface [110,111] or when −c<f<c [228], where c is an arbitrary numerical value that falls within the range of t, the level-set parameter; skeletal-based TPMS are created when f≥c or f≤−c (thus, there are two types of skeletal-based TPMS possible for a given TPMS surface type) [226,227,228]. For a more in-depth explanation with regards to the mathematical workings of the TPMS equations, the reader is directed to [226,227,228], while additional surface equations are documented in [229,230].

**Table 4:** Select equations from the literature for TPMS topologies. Sheet (left image) and skeletal (right image) illustrations of the TPMS topologies were created with MSLattice [206].

TPMS Type	Refs.	f(x,y,z)−C(x,y,z) ^1^
Diamond 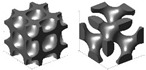	[110,205,210,229,230,231]	sin(kx)sin(ky)sin(kz)+sin(kx)cos(ky)cos(kz)+cos(kx)sin(ky)cos(kz)+cos(kx)cos(ky)sin(kz)−C
	[44]	sin(kx)sin(ky)sin(kz)+sin(kx)cos(ky)cos(kz)+cos(kx)sin(ky)cos(kz)+cos(kx)cos(ky)sin(kz)−0.07{cos(4kx)+cos(4ky)+cos(4kz)}−C
	[111,232]	sin(kx)sin(ky)sin(kz)+cos(kx)sin(ky)sin(kz)+sin(kx)cos(ky)sin(kz)+sin(kx)sin(ky)cos(kz)−C
	[233]	cos(kz)sin(kx+ky)+sin(kz)cos(kx−ky)−C
	[234]	cos(kx)cos(ky)cos(kz)−sin(kx)sin(ky)sin(kz)−C
Gyroid 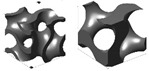	[204,205,210,231,233,234,235,236] In [112], C=1.6	sin(kx)cos(ky)+sin(ky)cos(kz)+sin(kz)cos(kx)−C
	[98,110,111,114,229,230,237,238,239]	cos(kx)sin(ky)+cos(ky)sin(kz)+cos(kz)sin(kx)−C
	[232]	cos(kx)sin(kx)+cos(ky)sin(ky)+cos(kz)sin(kz)−C
Schwarz P/Primitive 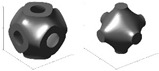	[111,112,114,205,210,215,229,230,231,232,233,237,239]	cos(kx)+cos(ky)+cos(kz)−C
Neovius 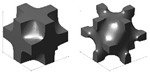	[210,215,229,230,231,240]	3{cos(kx)+cos(ky)+cos(kz)}+4cos(kx)cos(ky)cos(kz)−C
	[237]	cos(kx)+cos(ky)+cos(kz)+3cos(kx)cos(ky)cos(kz)−C
Split P 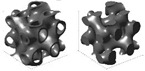	[210,229,230,231]	1.1{sin(2kx)sin(kz)cos(ky)+sin(2ky)sin(kx)cos(kz)+sin(2kz)sin(ky)cos(kx)}−0.2{cos(2kx)cos(2ky)+cos(2ky)cos(2kz)+cos(2kz)cos(2kx)}−0.4{cos(2kx)+cos(2ky)+cos(2kz)}−C
Lidinoid 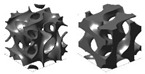	In [229,230,231], C=−0.3	sin(2kx)cos(ky)sin(kz)+sin(2ky)cos(kz)sin(kx)+sin(2kz)cos(kx)sin(ky)−{cos(2kx)cos(2ky)+cos(2ky)cos(2kz)+cos(2kz)cos(2kx)}−C
	[210]	0.5{sin(2kx)cos(ky)sin(kz)+sin(2ky)cos(kz)sin(kx)+sin(2kz)cos(kx)sin(ky)}−0.5{cos(2kx)cos(2ky)+cos(2ky)cos(2kz)+cos(2kz)cos(2kx)}−C
I-graph-Wrapped Package (IWP) 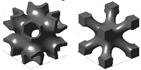	[101,112,118,215,234]	−2{cos(kx)cos(ky)+cos(ky)cos(kz)+cos(kx)cos(kz)}+{cos(2kx)+cos(2ky)+cos(2kz)}−C
	[114,240] In [229,230], C=−0.25	cos(kx)cos(ky)+cos(ky)cos(kz)+cos(kx)cos(kz)−C
Face-centered cubic Rhombic Dodecahedron (FRD) 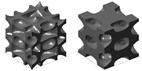	[112,229,230]	4cos(kx)cos(ky)cos(kz)−cos(2kx)cos(2ky)−cos(2kx)cos(2kz)−cos(2ky)cos(2kz)−C
Fisher–Koch C(Y) 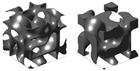	[234]	−sin(kx)sin(ky)sin(kz)+sin(2kx)sin(ky)+sin(2ky)sin(kz)+sin(2kz)sin(kx)−cos(kx)cos(ky)cos(kz)+sin(2kx)cos(kz)+sin(2ky)cos(kx)+sin(2kz)cos(ky)−C

^1^ Note: k=2π/L, where L is the length of a unit cell; C(x,y,z) is the isovalue function (for graded lattices) and is equal to constant t, the level-set parameter (overall range is dependent on the type of surface), for uniform lattices.

#### 4.3.3. Truss Topology Cross-Section Shape Design

**Figure 7:**
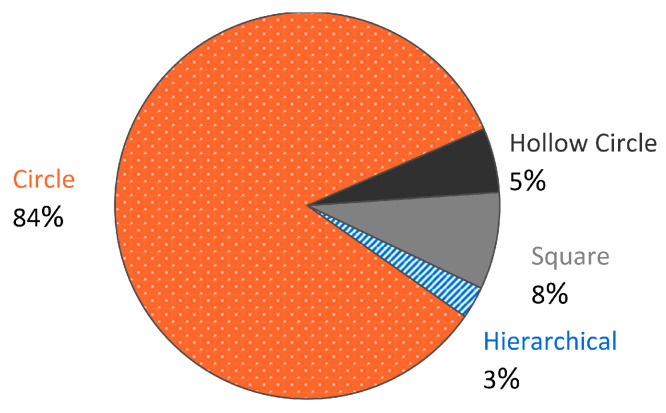
Distribution of cross-sectional shapes for strut-based topologies investigated in the literature. Slice for maximum value filled with orange speckle, slice for minimum value filled with blue diagonal lines, and all other slices filled in gray.

Perhaps limited due to the methods available for generating lattices, the vast majority of papers with truss-based topologies had those struts designed with circular cross-sections, as is illustrated in Figure 7. There are a few papers with non-hierarchical topologies that designed their topologies with non-circular cross-sections, namely [78,88,241,242,243,244,245], but they did not investigate the effect that any change in cross-sectional shape might have on the mechanical or energy absorption response of the lattice material. Since advantages to the properties of truss-based lattices for other applications have already been in development (specifically with the use of shape transformers, detailed by Pasini [246]), further research into this area for energy absorption capabilities could yield great results [55,247,248,249,250].

There have also been some papers that do not investigate the effect of changes to cross-sectional shape but instead the effect of non-constant diameter along the length of the struts [10,251,252,253,254]. In 2016, Tancogne-Dejean et al. [251] looked into the effect of varying the diameter of a circular cross-section over the length of a strut on the performance of an octet truss lattice under quasi-static and dynamic loading rates. They found that a ratio of minimum to maximum strut diameter of 0.8 provided the highest yield strength of the lattice material. Then, in 2018, Tancogne-Dejean et al. [252] derived analytical expressions for the homogenized macroscopic moduli of a BCC topology as a function of relative density and investigated the effects of strut tapering on the performance of a BCC lattice during quasi-static and dynamic loadings. They concluded that a BCC structure with tapered beams has higher specific mechanical properties than one with no tapering. Also in 2018, Cao et al. [10] investigated the effects of modifying the strut radius along its length for a rhombic dodecahedron topology. They introduced a shape parameter for the relationship of the minimum cross-section radius to the original (maximum) cross-section radius. It was possible to increase the compressive modulus, initial yield strength, and specific energy absorption (SEA) by modifying this relationship. Then, in 2020, Cao et al. [253] developed a finite element model where the lattice material model employed the Johnson–Cook strength and failure models. The results were compared to dynamic loading experimental results with good accuracy, and they found that at higher strain rates, the performance improved (from 0.001/s to 1000/s; higher compressive strength, plateau stress, normalized SEA). Using those experimental results, [254] validated a finite element model to further investigate the effect of the variation in cross-sectional area along the length of a strut on the performance of the rhombic dodecahedron topology. They also proposed a modified rigid, perfectly plastic-locking shock wave model, seeing good correlation between predicted results and FEA results.

Additionally, some hierarchical (or higher-order) topologies have been designed and investigated, where the zeroth-topology or first-order topology is generally a strut-based topology with circular cross-sections (e.g., an octet). Then, for higher-order hierarchical topologies, another topology is patterned along the struts of the lower-order topology (e.g., octahedron), such that on a macro-scale, the result is an octet topology, but on the meso-scale, there is the octahedron topology. Such an example is only for two levels of hierarchy (octet and octahedron) but can be continued for more levels. Lv et al. [171] investigated hierarchical lattices with the octet topology at the macro-scale. They noted that the peak stress and length of the stress plateau range for the second-order hierarchical octet (each rib of the re-entrant structure of the first-order cell replaced with a re-entrant hexagon, keeping strut aspect ratio constant) were smaller as compared to the first-order (each strut of the octet lattice replaced by the tubular re-entrant structure) and zeroth-order (each strut of the octet truss kept with a circular cross-section and the radius selected such that the relative density is the same as the hierarchical structures). However, its specific energy absorption was also lower than the first-order hierarchical lattice (but still higher than the zeroth-order). Bernal et al. [255] used the hierarchical design to develop a cellular material that attempted to combine the desirable effects of a periodic lattice structure with those effects of stochastic foams. By investigating this hierarchical structure (lattice macrostructure, foam microstructure), they found that the post-yielding flow stress was significantly increased as compared to only the lattice structure or only the stochastic foam, though they note that it is inconclusive as to whether the hierarchical concept allows for specific energy absorptions higher than simply the foam or lattice on its own.

While Lakes [256] pioneered the investigation of man-made hierarchical materials back in 1993 and while hierarchical structures have already begun to be applied to lattice materials for other characterization purposes [145,257,258,259,260,261], it is still lacking in the area of lattice materials for energy absorption applications.

#### 4.3.4. Lattice Cluster Design

When it comes to the selection of a single unit cell versus a cluster of unit cells (and the size/number of unit cells of that cluster), an important consideration is the manufacture time and build limits when manufacturing samples and the computation time when using finite element analysis. Larger samples may not be possible with the available additive manufacturing devices, and computation time can increase quickly as samples get larger (though element size within the model also plays an important role) [224,262]. Yet, accurately capturing the response of the lattice structure at this research stage is crucial, particularly since these cellular materials will generally be integrated into a component instead of being utilized in an assembly as a stand-alone cuboid volume [30,42,263,264,265]. Smith et al. [125] showed that it was possible to obtain mechanical properties from a numerical model containing one (or very few) unit cells. On the other hand, Morrish et al. [266] suggests a minimum of four unit cells along each orthogonal direction for the convergence of uniaxial compression results (note: they were working with cells with a cubic envelope and within the Cartesian coordinate system). Wang et al. [267] ran finite element simulations on clusters of 16, 100, 400, and 900 cells (note that this was for a single plane of cells (e.g., 16 cells = 4 cells × 4 cells) where the out-of-plane direction is orthogonal to the compression direction) and compared deformation modes between clusters. They noted that for the deformation mode of the 4 × 4 cluster, there was a greater boundary influence on the response and that both the 4 × 4 and 10 × 10 clusters had significantly different plateau stresses than the other clusters. For a balance between accuracy and computation time, they moved forward with the 20 × 20 cluster for further investigations.

Since most topologies within the papers of interest fit within a cubic representative volume envelope, clusters of cells for samples and within numerical models were generally tessellated along x-, y-, and/or z-directions, leading to the cluster fitting within a rectangular prismatic volume (i.e., a sample may have been replicated three times in the x-direction, three times in the y-direction, and four times in the z-direction, such that the cluster had an overall 3 × 3 × 4 arrangement of unit cells). Despite the cubic shape of those unit cells, however, some papers did investigate clustered samples with an overall cylindrical volume, not a rectangular prism. Wang et al. [238] investigated the behavior of a cluster of TPMS sheet gyroid cells arranged in a cylindrical shell shape. They began by proposing a method for mapping TPMS lattices to cylindrical shell specimen shapes before investigating the response of the gyroid cylindrical shell shape lattice numerically, validating by experiments. They noted that the energy absorption of such a lattice increased with increasing relative density and also investigated the effect of geometrical gradients on its compression response. Ahmadi et al. [17] experimentally investigated the mechanical behavior, including energy absorption capacity, of six topologies as cylindrical lattice samples. They found that the topologies could be divided between a group of high stiffness (truncated cube, truncated cuboctahedron, rhombicuboctahedron, cube) and of low stiffness (diamond, rhombic dodecahedron) topologies, also noting that the energy absorption of all topologies was similar across relative densities, except for diamond, which, at higher relative densities, had a much lower energy absorption as compared to the other topologies.

Additionally, configurations of lattices generally did not involve sandwich structures (i.e., with face sheets sandwiching the lattice as a core material), but there were some papers who did use the sandwich structure configurations. For example, Smith et al. [126] experimentally investigated the response of BCC and BCC-Z lattice clusters under quasi-static and blast loading conditions, finding that rate sensitivity could lead to a significant increase in yield strength at high strain rates. They also investigated the response of sandwich structures with carbon fiber-reinforced plastic face sheets and the lattice core, noting that the constraints imposed on the lattice by the face sheets improves the mechanical properties. Yazdani Sarvestani et al. [157] investigated the quasi-static bending and low-velocity impact response of sandwich panels with plate-based lattice cores. They noted that at a relative density of 30% (and larger unit cell) and under quasi-static bending, the panels with the plate-based octet topology had higher energy absorption than the other configurations. Yet, at a relative density of 50% (and smaller unit cell), the plate-based cubic and “Isomax” panels had higher energy absorption capabilities. They also noted that for impact, the three configurations had similar energy absorption capabilities. Shen et al. [123] investigated the compressive and bending responses of BCC and BCC-Z lattices, as well as the result of unit cell orientation with respect to load, under quasi-static and low-velocity loading. They found that topologies and orientations where there were struts aligned with the load direction had superior properties and that during low-velocity impact, the sandwich beams absorbed most of the impact energy directly at the point of impact. Gültekin and Yahşi [268] studied the crashworthiness of sandwich plates with lattice cores for application in battery housing. While they found that a plain sheet (no lattice core) and the 2D honeycomb designs led to the greatest energy absorption, the plain sheet also had the highest impact stress while the Kagome design had the lowest stress.

## 5. Characterization and Analysis Methods

**Figure 8:**
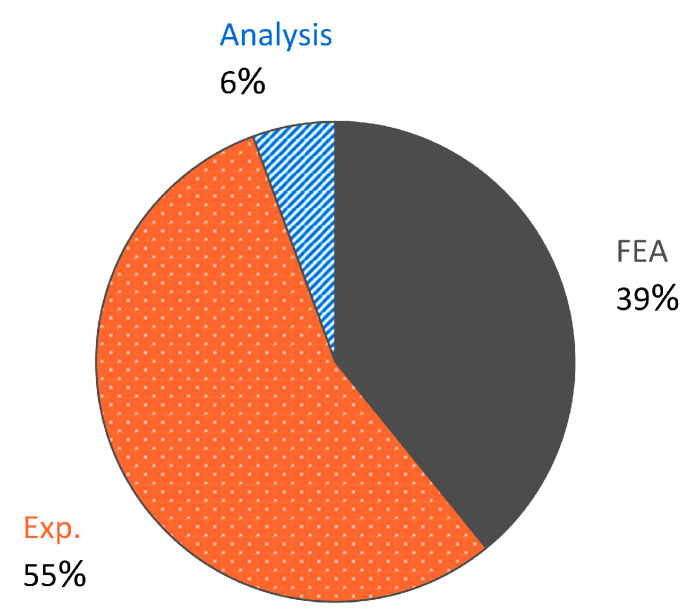
Distribution of methods for characterization. Slice for maximum value filled with orange speckle, slice for minimum value filled with blue diagonal lines, and all other slices filled in gray.

Bhate et al. in [109] describe three methods of approach for the selection of a topology for a given application: analytically, representing behavior using mathematical models; empirically, using experimental data to compare material behavior or to develop models for predicting behavior; or by optimization, using computational tools to derive a design based on multiple objectives. And, as was mentioned in [109], selecting a topology is usually approached through empirical (experimental or numerical) methods. Such an observation is reflected in the results of Figure 8, where over 90% of the different approaches taken are empirical and less than 10% are analytical in nature. It should be noted that if a paper used multiple approaches (e.g., FEA combined with experiments), both methods are counted; over 55% of papers used more than one approach. Additionally, while optimization (e.g., multi-objective, topology) is not listed in Figure 8, a handful of the papers did utilize this approach [43,112,159,160]; generally, such an approach was combined with FEA as well.

### 5.1. Loading Types

The vast majority of the literature reviewed focuses on compressive loading of the cellular materials, which is perhaps understandable given the focus of this paper on the energy absorption of those materials. However, during the high-level research stages while determining whether a paper did or did not investigate energy absorption, it was noted that compressive loading of cellular materials vastly outnumbers any other type of loading, such as tensile [99,269], bending [104,270,271], and fatigue [272,273,274], suggesting room for further investigation.

### 5.2. Experimental Test Standards

When it comes to the experimental testing of cellular materials, there are a couple recurrent testing standards utilized in the literature:

- ASTM D1621: Standard Test Method for Compressive Properties of Rigid Cellular Plastics [275];
- ISO 13314: Mechanical Testing of Metals—Ductility Testing—Compression Test for Porous and Cellular Metals [276], which are standards specifically for the experimental compression of cellular materials. However, as mentioned, some papers investigated the cellular materials as the core of sandwich materials, and additional testing standards were referenced for those unique tests [277,278]. For characterizing the parent material, usually for use within the material model for finite element analysis, other standards included the following:
- ASTM D638: Standard Test Method for Tensile Properties of Plastics [279];
- ASTM D695: Standard Test Method for Compressive Properties of Rigid Plastics [280];
- ASTM E8M: Standard Test Methods for Tension Testing of Metallic Materials [281];
- ASTM E9: Standard Test Methods of Compression Testing of Metallic Materials at Room Temperature [282].

For the most part, at least one applicable standard is referenced in a given paper, though it is not always the case. Additionally, while most of the papers referenced within this review deal specifically with additive manufacturing technologies, standards such as ASTM D638 and ISO 527-2 [283] (*Plastics—Determination of Tensile Properties—Part 2: Test Conditions for Molding and Extrusion Plastics*, the ISO near-equivalent of ASTM D638) were not specifically made with additively manufactured parts in mind. Though, perhaps interestingly enough ASTM ISO/ASTM 52921 [284]—which provides standard terminology for coordinate systems and test methods of AM technologies—cites the non-AM-specific ASTM D638, ASTM E8M, and ISO 527 standards. In 2015, Gao et al. [91] mention that the “rapid proliferation or AM technologies” has led to a lack of design guidelines and a lack of standard *best practices*. And in 2019, García-Domínguez et al. [285] reiterate that the prolific nature of the characterization of additively manufactured materials is not only a result of their increasing importance across industries, but also a lack of standardization. They reference Popescu et al. [286] who have specifically demanded for the development of test standards specific to FDM parts, and in [285], they compare the Spanish standard UNE 116005:2012 [287] to the 2014 version of the international standard ASTM D638, finding that the AM-specific national standard has better repeatability than the non-AM-specific international standard. It has been previously documented in [288] that ASTM D638 and ISO 527-2 produce similar results but do note the testing was performed over two decades ago using unfilled, unreinforced, and uncolored thermoplastic resin samples from a variety of laboratories, and those laboratories testing ASTM samples did not also test ISO samples. Additionally, the testing did not consider AM specimens. Furthermore, there are still critiques of the lack of a unified AM-focused standards, particularly as it pertains to the validity of the comparison of results between different sources, who may not have used the same machine, let alone the same numerous build parameters [286,289,290]. One could thus expand this critique of those ASTM and ISO standards to ASTM D1621 and ISO 13314, who are also not AM-specific.

### 5.3. Finite Element Method Solutions

When it came to finite element solutions for numerical modeling of lattice materials, the following were utilized:

- ABAQUS (generally Standard and/or Explicit) (Dassault Systèmes SE, Vélizy-Villacoublay, France) [291];
- LS-DYNA (Ansys Inc., Canonsburg, PA, USA) [292];
- RADIOSS (Altair Engineering Inc., Troy, MI, USA) [293];
- Ansys (generally Workbench) (Ansys Inc., Canonsburg, PA, USA) [294];
- DEFORM (Scientific Forming Technologies Corporation, Columbus, OH, USA) [295].

While finite element analysis is a good solution for costly experimentation (both in terms of time and money), it can itself also quickly become computationally expensive, especially for 3D elements or larger sample sizes [224,262]. Smith et al. [125] developed a finite element model to predict the response of BCC and BCC-Z lattice structures. They created two Finite Element (FE) models—one with 3D elements and another with beam elements (multiple collinear beams with different diameters—see Section 4.3 for more information)—and were able to obtain good agreement between the FE models and experimental results. While they did not investigate energy absorption characteristics, Ushijima et al. [296] developed a theoretical model for predicting the mechanical properties of the BCC lattice and compared it to experimental and FEA results. They noted the significance of the strut aspect ratio on the accuracy of predicting experimental results using either the theoretical or numerical approach. For aspect ratios above 0.1, the Finite Element Model (FEM) made of 1D beam elements was less accurate in predicting the experimental results; the FEM made of 3D solid elements agreed better with experiments over a larger range of strut aspect ratios.

Lattice samples with more unit cells can also increase computation time, making it desirable to determine the minimum number of unit cells to be able to accurately capture the response of lattices; Section 4.3 discusses this topic while Table 5 presents such advantages and disadvantages, including experimental testing and homogenization approaches. Though not a focus of this work and not a method utilized in the papers of interest, homogenization [297,298,299,300,301] as an approach to the investigation and characterization of lattice materials has already begun [140,302,303,304,305] and could prove to be a valuable avenue for this type of research as it can significantly reduce computation time in numerical models. An additional tool, which could help in the comparison of simulation data to experimental results and increase confidence in the accuracy of the simulation model, is EikoSim [306] and has been used in the successful vibration analysis of a hydromechanical actuator [307].

As previously mentioned in Section 4.3, finite element analysis also does not, by default, account for any geometrical imperfections that are present in as-built specimens, which can cause differences between the experimental and numerical results [124,223,308]. Lei et al. [124] manufactured BCC and BCC-Z lattice specimens using SLM technology and investigated the geometrical imperfections of the printed struts, using the X-ray micro-computed tomography (microCT) information to create an FE model that considered the non-uniformity of the struts based on the diameter of the beam elements within the model. They found that by incorporating these defects into the model, the results more closely matched the results of experiments, as compared to an FE model with a uniform cross-sectional area along the length of the struts. Tallon et al. [223] investigated the behavior of additively manufactured maraging steel rhombic dodecahedron lattices, specifically under quasi-static compression. They developed two numerical models to predict experimental results—one based on the as-built geometry following manufacture and the other based on the as-designed CAD geometry model. They found that the as-built geometry over-predicted the strength, possibly due to the model being unable to accurately model any micro-porosities and other additional complexities of an as-built sample, whereas the as-designed geometry model under-predicted the strength. This difference is mentioned to possibly be due to the specimen size used to calibrate the parent material tensile response in the numerical model (the struts being only one tenth the size of the specimen used for calibration). They also highlight that the as-designed geometry model has some additional stress concentrations where struts join; in the as-built model, as a result of the additive manufacturing process, the struts are joined with something resembling fillets, reducing the concentration of stresses in those areas. While they do not investigate energy absorption characteristics, Bahrami Babamiri et al. [308] reconstructed the as-printed geometry model from X-ray computed tomography (XCT) as well and used this model within finite element analyses, comparing to experimental results and numerical results with an as-designed CAD geometry model. They found that the stress–strain results of the finite element model based on the XCT geometry predicted experimental results better than the as-designed CAD geometry model, which did not account for any strut non-uniformity or surface roughness. Using this observation, they calculated correction factors based on the ratio of the relative density of the as-built sample to the as-designed CAD model.

As from Section 5.1, there are applicable testing standards for the characterization of parent material properties, which can be used to define material models within numerical models or used for analytical investigations. For the most part, these standards require a dog-bone-shaped specimen where, for example, an ASTM D638 Type I specimen has overall dimensions of 3.2 mm × 19 mm × 165 mm (thickness × width × length) [279]. Yet, as briefly mentioned previously, this scale can be orders of magnitude larger than the scale of the components of a topology (e.g., diameters of struts, thicknesses of surfaces). Outside of the realm of the papers identified as focusing on energy absorption properties, there has been some material testing on long, cylindrical strut-like samples whose diameters are more representative of the scale of the struts of the lattice topology. Tsopanos et al. [89] printed individual struts at different angles relative to the build direction and performed tensile tests to characterize their response for the use in analytical and numerical models, finding reductions in yield stress as the strut angled further from vertical. Li et al. [309] ran tensile tests on struts with diameters of 1 mm and 5 mm (where the struts within the BCC-Z lattices had diameters of 1 mm), finding that the mechanical results of the larger sample were higher than those results from the smaller sample, attributing the differences to the effects of temperature during the powder-based SLM process, where the smaller sample would have had less accumulation of heat, leading to some un-melted powder particles. Gümrük and Mines [310] used the stress–strain results from an individual strut within theoretical and numerical studies, noting it could be a practical and efficient way to incorporate defects into those models (as the material results more closely resemble the as-built struts, and there is no need to attempt to model the complex surface of the lattice, which differs from the as-designed CAD model by its very nature).

Another point to note on the topic of standards and finite element method solutions is the general lack of consistency in terms of boundary conditions, initial conditions, and general variations in numerical model set-up between different publications. Such variations in the finite element approach, as critiqued by Helou and Kara [53], make results difficult, or impossible, to compare between sources, and they suggested, already a few years ago now, “the creation of a detailed FEA procedure identifying and clarifying boundary conditions specific to lattice structures.” Additionally, it has already been noted in Figure 6 that most publications only investigate one topology, which further limits the accurate comparison of performance between topologies.

## 6. Impact Strain Rates and Impactor Shapes

### 6.1. Speeds and Strain Rates

**Figure 9:**
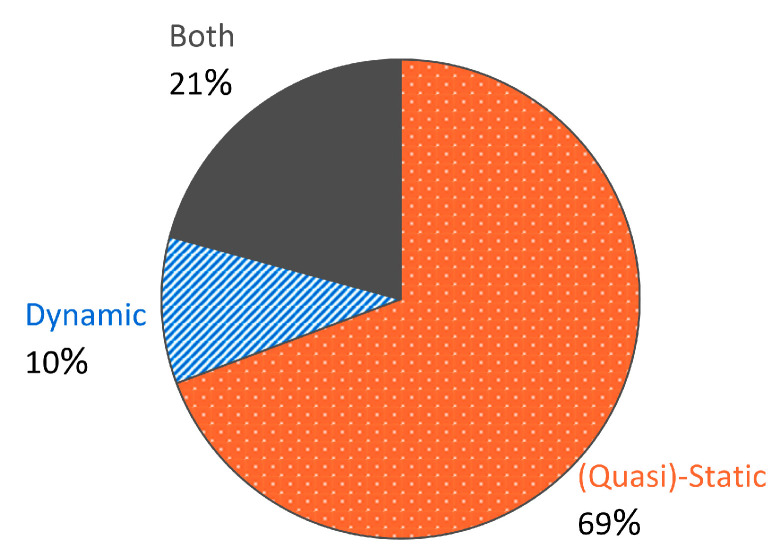
Distribution of the literature investigating (quasi-)static versus dynamic loading rates. Slice for maximum value filled with orange speckle, slice for minimum value filled with blue diagonal lines, and all other slices filled in gray.

As is evident in Figure 9, the majority of the literature reviewed focuses solely on the quasi-static response of lattice materials. Additionally, 21% still investigate the quasi-static response, just in conjunction with dynamic loading. As mentioned in Section 1, an application of the energy absorption properties of lattice materials is for crash/blast protection, protective packaging, and within contact sports helmets. Such applications will generally enter, at the very least, the low dynamic loading range, with the possibility of high dynamic, or even ballistic/blast speeds.

For those papers that did venture into the experimental dynamic strain rate range, typical equipment used included drop (weight) towers [70,119,123,133,154,157,235,311,312], (Split) Hopkinson Pressure Bars (S/HPBs) [102,129,235,245,251,252,253], or ballistic pendulums [119,126]. The cost for such equipment, particularly S/HPBs, can quickly become prohibitive in nature, perhaps suggesting one reason for the majority of the literature focusing instead on more accessible testing [313,314]. Additionally, S/HPBs generally require a large footprint, which may not be possible for all labs to accommodate, and while some smaller devices do exist, such as the *desktop Kolsky bar*, those smaller devices would also limit the maximum specimen dimensions [315]. Digital Image Correlation (DIC) may also be utilized for the measurement and characterization of these materials under dynamic loading, which would require additional equipment, increasing the experimental cost [47,187,253,316,317,318,319].

### 6.2. Impactor Shapes

Across the reviewed literature, whether for quasi-static or dynamic loading, the majority of the compression plate or impactor shapes were flat where they contacted the lattice materials with few exceptions, and, of those that did, there was not an investigation of the effect of other impactor shapes. Epasto et al. [311] investigated the performance of titanium alloy rhombic dodecahedron lattices manufactured via EBM under both quasi-static and impact loading conditions. They noted that by decreasing the cell size, the compression and crush strength increased, while the specific energy absorption was at its optimal point with the mid-cell size. They also performed some heat treatments on as-built lattices, testing their compression response and noting that the performance actually decreased following heat treatment, attributing this result to the insignificant effect that residual stresses (if present) play in the response of as-built specimens. They also concluded by noting that a smaller unit cell size, but higher relative density, should be utilized in crashworthiness applications.

While they did not focus on energy absorption properties of lattices specifically, Mines et al. [312] experimentally investigated the impact performance of sandwich panels with Carbon Fiber-Reinforced Polymer (CFRP) skins and BCC cores, manufactured using SLM out of either Ti6Al4V or SS316L. They noted the influence of processing parameters on the quality of the manufactured specimens, finding that the SS316L BCC cores were less sensitive to those variables and had a higher build quality but had lower specific strength as compared to the Ti6Al4V ones. In comparison to aluminum honeycomb sandwich panels, the Ti6Al4V ones were noted as capable of competing with their performance.

**Figure 10:**
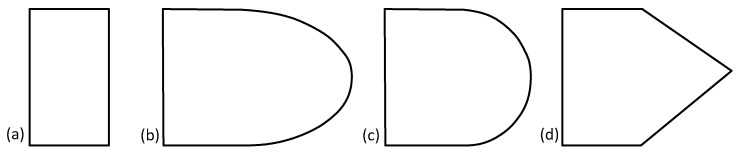
Common profiles of impactors. (**a**) Flat, (**b**) ogival, (**c**) hemispherical, and (**d**) conical.

Other research areas have begun to investigate performance based on impact with non-flat shapes and impactors of various sizes [320,321,322,323,324,325], with some common profiles illustrated in Figure 10, and they were found to influence the energy absorbed and peak force during impact. As investigations into the energy absorption performance of materials based on impact by non-flat (e.g., spherical, conical) shapes are being accomplished in other areas and would begin to represent more realistic impact conditions, such an avenue is sensible for lattice materials as well.

## 7. Energy Absorption Trends in the Literature

In addition to the qualitative data collected whose trends have been discussed in the previous sections, quantitative data for the energy absorption results from the 100 papers of interest were collected (as available) and analyzed for trends. Such data were collected directly from tabulated results or from images using the online WebPlotDigitizer (Automeris LLC, CA, USA) [326], a tool for extracting numerical data from graphs and plots. It is noted that the latter method does inherently introduce some uncertainty in the numerical values extracted but it has been noted to be an acceptable method, particularly for allowing for the analysis of *trends* and *not exact numerical values*. Hanks et al. [327] utilized the WebPlotDigitizer during the collection of a variety of numerical mechanical properties (Young’s modulus, yield, strength, Poisson’s ratio, etc.) for their Lattice Unit-cell Characterization Interface for Engineers (LUCIE, PennState, University Park, PA, USA [328]), an application that compiles (to date) the mechanical property data for 18 topologies from 69 papers, creating Gibson–Ashby plots to aid in the selection of a unit cell topology during design.

Within this section, specific energy absorption by volume ($SEA_{vol}$) versus part density is plotted for those papers of interest. Lines of continuous specific energy absorption by mass ($SEA_{m}$) are plotted to aid in data visualization. Once the energy absorption data were plotted, four categorical highlights were made to observe trends: material type (as in Section 3.1); manufacturing method (as in Section 3.2); and topology (as in Section 4.1; divided into truss-based as in Figure 4 and TPMS as in Figure 5). There are also two figures that plot $SEA_{m}$ versus relative density to further aid in the comparison of truss-based and TPMS topologies, respectively. It should be noted that

**Figure 11:**
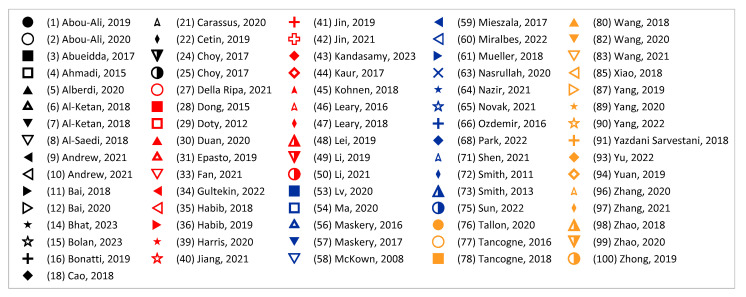
Legend for symbols in remaining figures of this section. Number in bracket refers to paper as listed under “Num.” column in Table A1, which can be used to locate the direct reference in question.

- Not all papers of interest had adequate data for collection and presentation. Common reasons as to why data could not be collected from a source include unit ambiguity for reported energy absorption or specific energy absorption values and/or failing to explicitly provide either relative density or part density (not to be confused with parent material density). In the end, the data from 76 papers were presented in each of the figures of this section.
- If a paper did not explicitly state the parent material density, a default value was utilized in conjunction with the relative density to calculate part density, a selection of which is provided in Table 6.
- For functionally graded lattices, data were plotted for the average part density.
- While the *y*-axis data are specific energy absorption per volume, it should be noted that the strain point for calculating these values did and does vary across the literature (e.g., at the densification strain, at 30% strain, at 60% strain).
- The legend for the figures in the following subsections is provided in Figure 11 due to the large number of sources. Each entry is provided a number in brackets, which can be cross-referenced to Table A1 for the actual numerical reference.
- These graphs can be utilized to identify gaps in the literature, highlighting areas of future research while also being used as a tool during the design selection process. However, as previously discussed in Section 5.1, the test standards utilized are not all the same across the literature; the reader should be aware of potential differences in test configurations while analyzing the presented graphs.

**Table 6:** Selection of default parent material density [kg/m^3^] used when not explicitly stated in the source paper.

Aluminum (AlSi10Mg)	Steel (SS316L)	Titanium (Ti6Al4V)	Nylon (PA12)
2670 [122]	8000 [119]	4430 [329]	919 [143,144]

### 7.1. Material Type Trends

**Figure 12:**
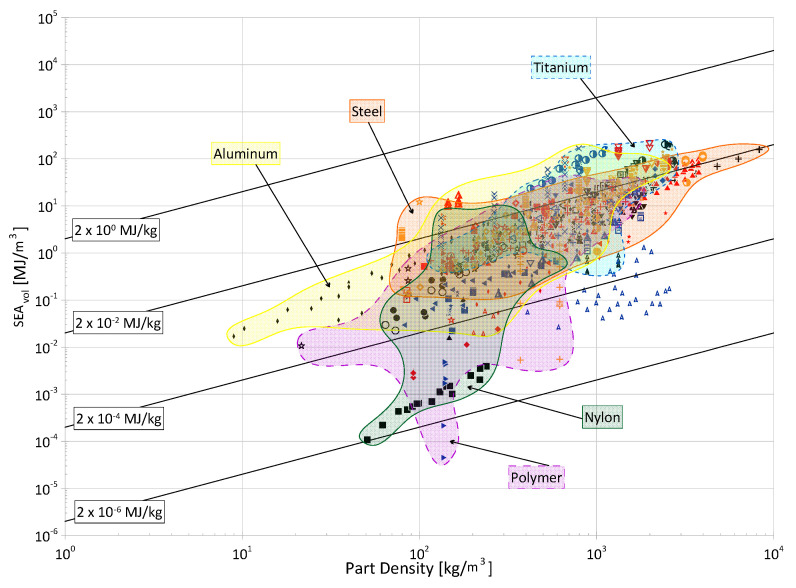
Specific energy absorption by volume versus lattice part density with highlights for select categories of parent material types. The “Polymer” group includes PLA, ABS, etc., but excludes nylon.

Based on the results from Figure 2, those material categories with over ten instances within the investigated papers (aluminum, steel, titanium, polymer/resin, and nylon) were plotted, as in Figure 12. Note that while nylon is itself a polymer, it was utilized enough as a parent material to warrant a separate category for analysis.

As in Figure 12, the metals—aluminum, steel, and titanium—generally trend to higher specific energy absorption per mass ($SEA_{m}$) values ($2\times 10^{-4\;}\mathrm{MJ}/\mathrm{kg}$ to higher than $2\times 10^{-2}\;\mathrm{MJ}/\mathrm{kg}$) while it is possible for the nylon and other polymers to fall below $2\times 10^{-6}\;\mathrm{MJ}/\mathrm{kg}$. However, it appears possible to increase the relative density (and thus the part density) of any part—regardless of parent material—above approximately $10^{2}\;\mathrm{kg}/m^{3}$ to achieve an $SEA_{m}$ greater than $2\times 10^{-2}\;\mathrm{MJ}/\mathrm{kg}$.

### 7.2. Manufacturing Method Trends

**Figure 13:**
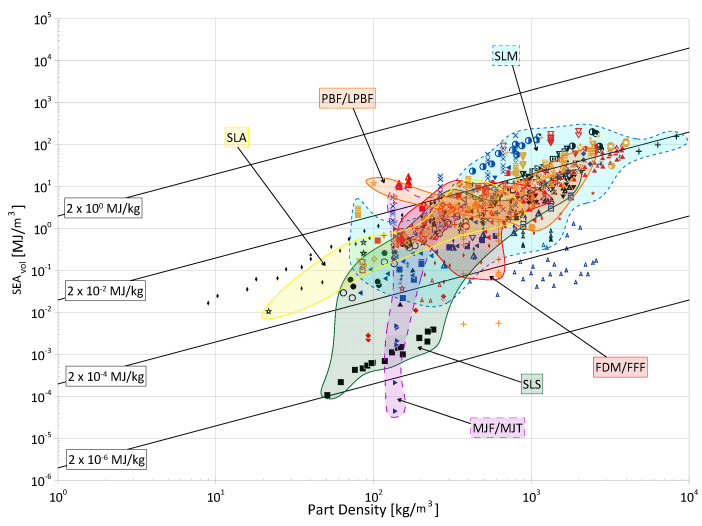
Specific energy absorption by volume versus lattice part density with highlights for select categories of manufacturing methods.

Based on the results from Figure 3, the manufacturing method categories with counts of greater than five (FDM/FFF, MJF/MJT, PBF/LPBF, SLM, SLS, and SLA) were plotted, as in Figure 13, with the exception of the “Other” category.

In combination with Figure 12, it is possible to see the relationship between material and manufacturing method in Figure 13. SLA, MJF/MJT, and SLS utilized nylon, other polymers, and resin and thus result in the same $SEA_{vol}$ and $SEA_{m}$ results as those parent materials. On the other hand, metal parts from aluminum, titanium, and steel were manufactured using powder bed fusion techniques (PBF/LPBF, SLM), and the overall energy absorption trends reflect it.

### 7.3. Truss-Based Topology Trends

**Figure 14:**
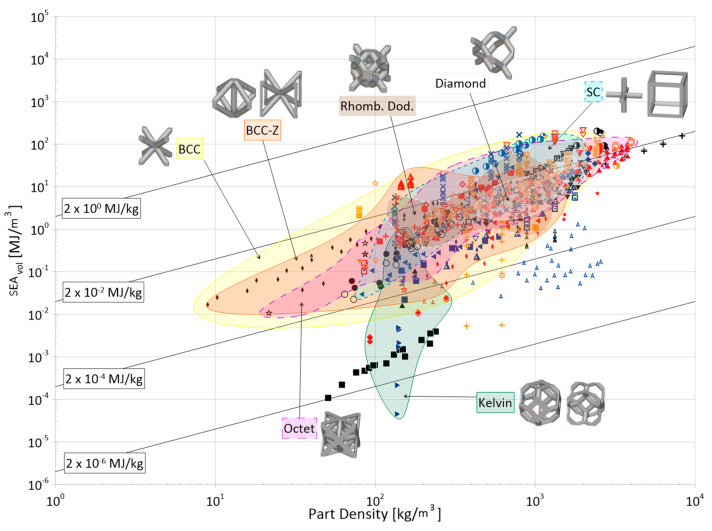
Specific energy absorption by volume versus lattice part density with highlights for select categories of truss-based topologies. TPMS topology highlights shown in Figure 16 for these axis variables.

**Figure 15:**
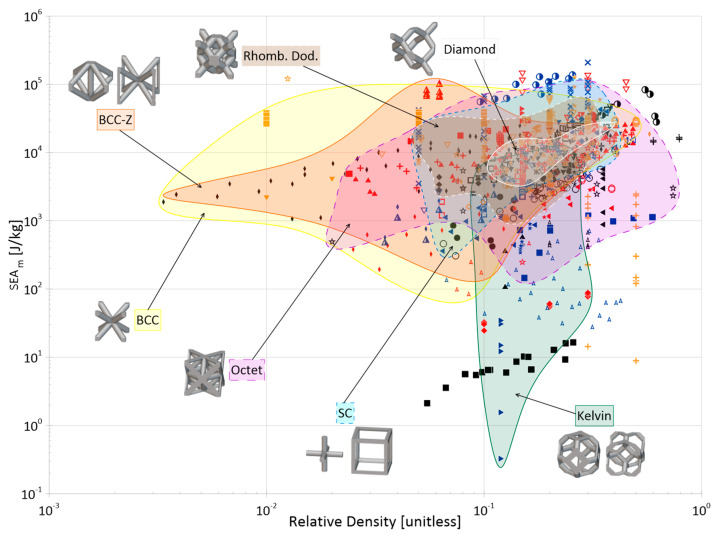
Specific energy absorption by mass versus lattice relative density with highlights for select categories of truss-based topologies. TPMS topology highlights shown in Figure 17 for these axis variables.

For truss-based topology trends, those topology categories with over five instances, per Figure 4, (BCC, BCC-Z, cubic (simple cubic, “SC”), diamond, Kelvin, octet, rhombic dodecahedron) are plotted, as in Figure 14, with the exception of the “Other” category. Those same categories were also plotted for $SEA_{m}$ versus relative density in Figure 15, which further aids in comparing and contrasting the topologies.

From Figure 14, it appears that there is no obvious difference between the bending-dominated BCC, BCC-Z, cube, diamond, and rhombic dodecahedron [10,120,121,129,134,150,160,241,245,254,330] and the stretching-dominated octet [129,134,138,146,160]; based on variations in the parent material and relative density of the lattice, the $SEA_{m}$ generally falls between $2\times 10^{-4}\;\mathrm{MJ}/\mathrm{kg}$ and $2\times 10^{-2}\;\mathrm{MJ}/\mathrm{kg}$. It is notable that at lower part densities ($<\sim 10^{2}\;\mathrm{kg}/m^{3}$), the range of $SEA_{m}$ for the octet topology begins to fall to the lower part of the range of the BCC and BCC-Z energy absorptions. Additionally, the bending-dominated Kelvin [134,143,146,150] generally absorbs energy per mass at a couple of magnitudes less than the other topologies highlighted ($2\times 10^{-6}\;\mathrm{MJ}/\mathrm{kg}$ to $2\times 10^{-4}\mathrm{MJ}/\mathrm{kg}$). It is also interesting that, of the papers for which these data were presented, there was a wide range of part densities (and, thus, relative densities) investigated for BCC, BCC-Z, and octet, with slightly fewer for cubic, rhombic dodecahedron, and diamond, and the fewest for Kelvin; while the data of the former six topologies would create a linear trendline with a positive slope, the Kelvin topology data would have an almost-vertical trendline.

From Figure 15, this observation regarding the span of relative densities investigated is further illustrated; the Kelvin topology generally did not get investigated at relative densities below 0.1 while BCC and BCC-Z topologies have data from relative densities as low as approximately $3\times 10^{-3}$.

### 7.4. TPMS Topology Trends

**Figure 16:**
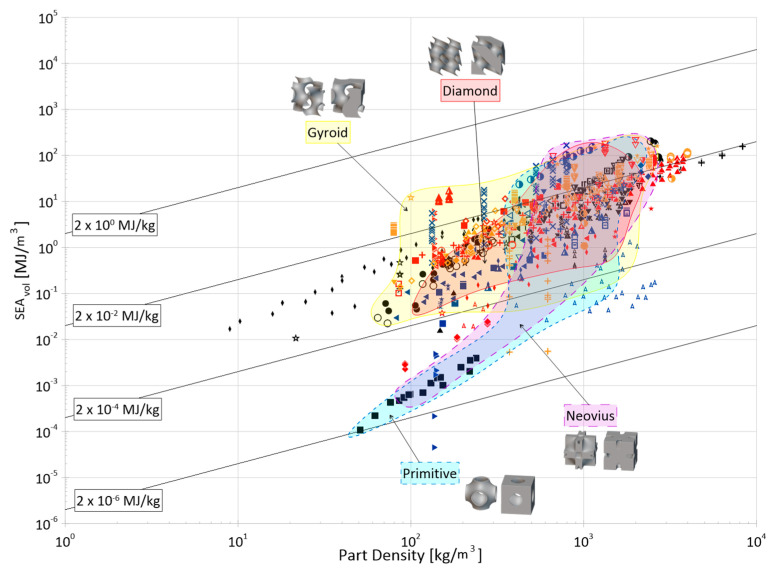
Specific energy absorption by volume versus lattice part density with highlights for select categories of TPMS topologies. Truss-based topology highlights shown in Figure 14 for these axis variables.

**Figure 17:**
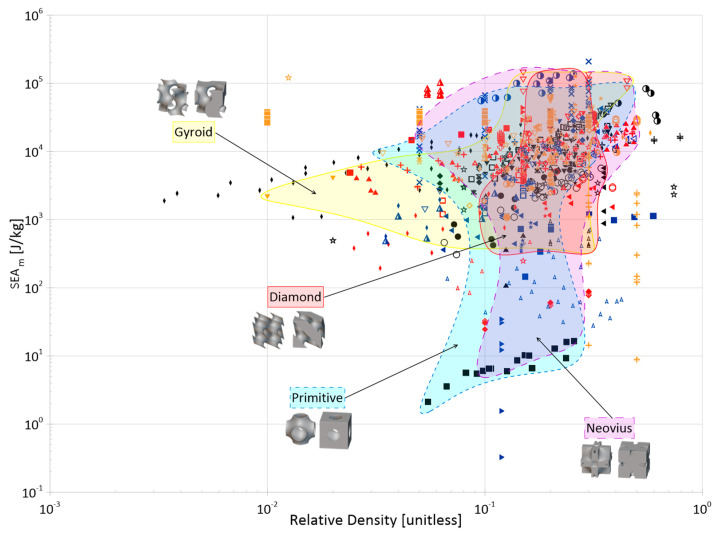
Specific energy absorption per mass versus lattice relative density with highlights for select categories of TPMS topologies. Truss-based topology highlights shown in Figure 15 for these axis variables.

For TPMS topology trends, the diamond, gyroid, primitive, and neovius categories were plotted, as in Figure 16, as they all had five or more instances within the literature per Figure 5. Figure 17, which plots $SEA_{m}$ versus relative density for those same categories, has also been presented to further aid in the comparison of these TPMS topologies. It should be noted that there is no distinction between sheet and solid variations in these TPMS topologies within these figures (i.e., the gyroid category contains both sheet and solid versions of the gyroid topology).

From Figure 16, at part densities above about $4\times 10^{2}\;\mathrm{kg}/m^{3}$, the gyroid, diamond, primitive, and neovius TPMS topologies all have similar ${\mathrm{SEA}}_{\mathrm{vol}}$ ($>10^{-1}\;\mathrm{MJ}/m^{3}$) and ${\mathrm{SEA}}_{m}$ ($>2\times 10^{-4}\;\mathrm{MJ}/\mathrm{kg}$). Below this part density value, the gyroid and diamond TPMSs continue to perform above ${\mathrm{SEA}}_{m}>2\times 10^{-4}\;\mathrm{MJ}/\mathrm{kg}$, but the primitive and neovius TPMSs drop, ending near ${\mathrm{SEA}}_{m}≅2\times 10^{-6}\;\mathrm{MJ}/\mathrm{kg}$ at a part density below $10^{2}\;\mathrm{kg}/m^{3}$.

From Figure 17 it is seen that the gyroid TPMS topology is examined at the largest range of relative densities, whereas the diamond, primitive, and neovius TPMS topologies only reach minimum relative densities of about $3\times 10^{-2}$. Interestingly, as a relative density between about 0.1 and 0.3, it is possible to manipulate the design to have any one of the four TPMS topologies yield the same $SEA_{m}$ as the others.

## 8. Conclusions and Outlooks

A review of the literature from 100 papers from approximately the last two decades was performed, which focused specifically on 3D periodic cellular materials for energy absorption applications. The process of collecting qualitative and quantitative data from those papers and presenting the data in Table A1 in multiple graphs has allowed for trends and gaps to be identified and suggestions for future avenues of advancement and research to be provided. Keeping with the organization of the paper, the following paragraphs summarize these trends and the suggestions for growth opportunities within this field of research.

*Material Types, Manufacturing Processes, and Post-Processing Treatments:* Materials are dependent on the availability of technologies for manufacturing the complex geometries, but pushing the limits of the available technology (e.g., by using an “uncommon” material and developing manufacturability parameters) could yield interesting and valuable results. Additionally, further advancement in AM techniques will only allow for further advancement in the area of cellular and lattice material manufacture. Heat or chemical treatments to modify the microstructure or roughness of strut-based lattices may be required to ensure anticipated and consistent manufacture quality.

*Topology Classification:* Topology naming is sometimes inconsistent in the literature, and with the introduction of new topologies, either through multi-objective or topological optimization or as bio-inspired geometries, a robust classification and naming system could help ensure better consistency and clarity when it comes to obtaining past research and presenting current research on a given topology; defining new topologies versus combinations or variations in existing topologies; and defining cellular materials based on characteristics such as cell symmetry, hierarchy, and multiple materials.

*Topology Design Software:* Referencing the type of design software utilized is not the norm within the literature, making it difficult to determine a robust and flexible program that has the potential to generate a wide variety of topologies, either as unit cells or as clusters. Even more lacking is the knowledge of which programs could be used to design hierarchical lattices or lattices with non-circular or non-uniform cross-sections. Without access to such technology, the collaborative research effort is limited.

*Truss Topology Cross-Section Shapes:* There are very few pieces of literature that investigate strut-based lattices with anything other than a uniform cylindrical strut. Research to-date suggests such changes are beneficial for mechanical and energy absorption properties, indicating further research could be valuable.

*Experimental Test Standards:* There are currently no standards developed with additively manufactured materials in mind, making the repeatability and comparison of AM materials difficult, particularly given the variety of process parameters that could be varied during manufacture and that have an effect on the final performance of the part. Additionally, the variety of initial conditions, boundary conditions, and general numerical model set-ups can make result comparison between the literature difficult, if not impossible.

*Test Strain Rates:* The majority of the investigated literature explores only a quasi-static strain rate response of the lattice material. With the knowledge of the energy absorption capabilities of lattice materials, higher strain rates can yield additional information about the types of applications these lattices, and given topologies, would be best suited for.

*Impactor Shapes:* Generally quasi-static and impact testing is performed such that the surface contacting the cellular material specimen is flat. Further investigation into the variation in performance under impact with non-flat surfaces could yield interesting energy absorption and deformation behavior results.

*Energy Absorption Trends:* Due to some challenges encountered in extraction, in the quantitative energy absorption data from all 100 papers of interest, only 76 had acceptable data for plotting. Based on those results, the following are noted trends, though the graphs could also be referenced to aid in the preliminary design process:

- Lattices manufactured from aluminum, steel, and titanium tend to have higher $SEA_{vol}$ and $SEA_{m}$ as compared to nylon and other polymer or resin parts, though there is a range of part densities where those results are comparable between metals and non-metals.
- The additive manufacturing techniques of SLA, PBF/LPBF, and SLM have similar $SEA_{m}$ results, while MJF/MJT and SLS tend to result in lower $SEA_{m}$ and $SEA_{vol}$ energy absorption performance.
- The bending- and stretching-dominated topologies examined had similar energy absorption results, though the Kelvin cell had a notably lower minimum $SEA_{m}$ than other strut-based topologies.
- At higher part densities, the gyroid, diamond, primitive, and neovius TPMS topologies all performed similarly in terms of $SEA_{vol}$ and $SEA_{m}$ results; at lower part densities, the gyroid and diamond topologies still had similar performances, but the $SEA_{m}$ and $SEA_{vol}$ for the primitive and neovius topologies dropped by a couple of orders of magnitude.
- In examining $SEA_{m}$ versus relative density, it became apparent which topologies were investigated at a wider range of relative densities; the Kelvin cell had no data below a relative density of about 0.1, while the BCC and BCC-Z topologies had data collected from as low as about 0.003. For the TPMS topologies, the gyroid had the largest range investigated, while the neovius and primitive barely went below 0.04 and diamond was only to a minimum of about 0.1.

## Figures and Tables

**Table 3:** Non-exhaustive list of software available for lattice geometry generation.

Software	Description	Utilized in
Altair Inspire (Altair Engineering Inc., Troy, MI, USA) [184]	has a PolyMesh module that provides the capability to fill a volume with a lattice mesh, selecting from a library of topologies [185]	[160]
Ansys SpaceClaim (Ansys Inc., Canonsburg, PA, USA) [179]	can fill a selected part with one of over ten topologies by making use of the “Facets” tab [186]	[187] Ref. [141] developed a script to automate the geometry generation with a library of 15 strut-based topologies and different cross-sectional shapes
Autodesk Fusion 360 (Autodesk Inc., San Francisco, CA, USA) [188]	has a Volumetric Lattice tool to fill a selected body, with the ability to select from a library of unit cells (as well as create a custom unit cell based on selected geometry) [189]	[190]
CATIA (Dassault Systèmes SE, Vélizy-Villacoublay, France) [191]	-	[192] Ref. [183] manually created topology within CATIA
CUBIT (Sandia National Lab, Albuquerque, NM, USA) [193], coreform Cubit (Coreform LLC, Orem, UT, USA) [194]	-	[130]
Materialise’s 3-matic (Materialise LV, Leuven, Belgium) [195]	has a Lattice Module for internal or external lattice structure design [195]	[196,197]
MATLAB (MathWorks, Natick, MA, USA) [198]	has multiple open-source tools and programs that aid in the geometry generation of lattices: TPMS Designer [199] described in [200] as “a tool for rapidly generating, visualizing and analyzing implicitly defined structures” with the ability to export to traditional CAD programs STL Lattice Generator [201] described in [202] as “a highly customisable free open source method of generating periodic lattice structures directly to the generic STL format”	[101,110,111,114,203,204,205]
MSLattice (NYU Abu Dhabi, Abu Dhabi, United Arab Emirates) [206]	detailed in [207] as “a software that allows users to design uniform, and functionally [graded] lattices and surfaces based on TPMS using two approaches, namely, the sheet networks and solid networks”	[208]
nTopology (nTop, New York City, NY, USA) [178]	utilizes an implicit approach to modeling strut and TPMS lattices, with a library of over 30 topologies (strut-based, TPMS, plate-based, etc.) and the ability to add more [178,209]	[107,109,210,211]
Rhinoceros (Robert McNeel & Associates, USA) [212] and its graphical algorithm editor Grasshopper (Robert McNeel & Associates, USA) [213]	has multiple plugins providing the capacity to design lattice structures: Crystallon [214] IntraLattice [153]	[215] Ref. [150] developed a lattice structure generator plugin for Rhinoceros with a library of topologies to choose from Ref. [216] utilized, among other commands, CreatePipe in Rhino/Grasshopper to create the lattice based on a mesh from ABAQUS (Dassault Systèmes SE, Vélizy-Villacoublay, France)
SolidWorks (Dassault Systèmes SE, Vélizy-Villacoublay, France) [217]	-	[78,101,115,124,133,218,219] Refs. [216,220] utilized SolidWorks’ Application Programming Interface (API) to create a library of unit cells to select from when generating a lattice within the design space

**Table 5:** Advantages and disadvantages of experimental and simulation approaches. Adapted from [224], additionally referencing [80,181].

Approach	Advantages	Disadvantages
Experimental	Reflects as-fabricated properties Can be used to validate simulation results	High cost for manufacturing Geometry will differ from CAD model, could have defects, and may require post-processing to eliminate Standard test machines may not be suitable for complex components
Homogenization	Low computational cost Can be used to represent lattice material in multi-material hybrids	Not applicable to heterogeneous lattices (e.g., functionally graded) Not easy to incorporate manufacturing defects Mathematically difficult to implement on new topologies
Finite Element (2D beam elements)	Low computational cost Can model heterogeneous lattices, irregular strut thickness (variations in beam diameter, stiffness)	Using a beam element requires assuming slender strut Does not model the manufacturing defects Cannot accurately model the joint geometry
Finite Element (3D solid elements)	Can use an as-fabricated model by X-ray or microCT image to accurately capture the as-fabricated geometry Models to joint geometry	High computational cost Difficult to mesh a thin strut Dependent on mesh quality

## Data Availability

Data are contained within the article.

## Appendix A

**Table A1:** Summary of methods, materials, cell parameters, and loadings for papers of interest. Acronym definitions are provided at bottom of table. “Num.” column refers to symbol type as listed in Figure 11 for Figure 12, Figure 13, Figure 14, Figure 15, Figure 16 and Figure 17.

Num.	First Author and Reference	Year	Method	Material	Relative Density	Cell Size	Single/Cluster	Topology	Cross-Section	Manuf. Process	Software	Loading Type	Rate Type	Strain Rate/Speed
1	Abou-Ali [234]	2019	E	PA1102	0.061–0.254	8 mm	Cl (5 × 5 × 5)	TPMS skeletal (Diam, IWP, Gyr, Fisher–Koch C(Y))	-	SLS	-	C	S	0.001/s
2	Abou-Ali [117]	2020	F, E	PA1102	0.061–0.254	8 mm	Cl (3 × 3 × 3, 5 × 5 × 5)	TPMS (Sheet: Schwarz Diam, Schoen Gyr, Schoen IWP; Skeletal: Schoen Gyr, Schoen IWP)	-	SLS	Ab	C	S	0.1/s, 0.01/s, 0.05/s, 0.001/s, 0.0001/s
3	Abueidda [331]	2017	F, E	PA2200	0.048–0.256	1.5 mm	Single, Cl (2 × 2 × 2, 4 × 4 × 4)	TPMS (Schwarz Prim, Schoen IWP, Neo)	-	SLS	Ab	C	S	0.001/s, 0.01/s, 0.1/s
4	Ahmadi [17]	2015	E	Ti6Al4V ELI	0.06–0.37	1.5 mm	Cl (cyl. ~7 × 10)	SC, Diam, Tr-SC, R-C-Octah, R-Dod, Tr-C-Octah	Ci	SLM	-	C	S	1.8 mm/min
5	Alberdi [130]	2020	E	SS316L, Vero White	0.125,0.15	4 mm, 8 mm	Cl	BCC, octet	Ci	LBF, MJT	-	C	S	5 × 10^−3^/s
6	Al-Ketan [118]	2018	E	Maraging steel	0.071–0.22	7 mm	Cl (6 × 6 × 6)	TPMS sheet and skeletal (IWP), BCC	Ci	SLS	-	C	S	0.001/s
7	Al-Ketan [115]	2018	E	Maraging steel	0.05–0.25	7 mm	Cl (6 × 6 × 6)	Kelvin, octet, Gibson–Ashby, TPMS (sheet: IWP, Prim, Gyr, Diam; skeletal: Diam, IWP, Gyr)	Ci	PBF	-	C	S	0.001/s
8	Al-Saedi [142]	2018	F, E	AlSi-12	FG (0.185 avg)	5 mm	Cl (6 × 6 × 6)	FBCC	Ci	SLM	LS	C	S	0.05 mm/s
9	Andrew [154]	2021	E	PlasGRAY	0.25–0.55 (constant thck. 1 mm)	16 mm	Cl (2 × 2 × 2)	(Plate-based) SC, BCC, FCC, SC-BCC, SC-FCC, SC-BCC-FCC (rotation of unit cell)	-	SLA	-	C	D	1.54 m/s to 3.09 m/s (20 to 80 J)
10	Andrew [70]	2021	E	HDPE, PPR, HDPE/MWCNT, PPR/MWCNT	0.36	Unc. (appears to be 16 mm)	Cl (2 × 2 × 2)	(Plate-based) SC, BCC, FCC, SC-BCC, SC-FCC, SC-BCC-FCC	-	FFF	-	C	D	1.5 m/s to 4.2 m/s (20 to 150 J)
11	Bai [332]	2018	F, E, A	Ti6Al4V	0.26	4 mm	Cl (8 × 8 × 8)	BCC, AFCC	Ci	SLM	Ab	C	S	1 mm/min
12	Bai [333]	2020	F, E	PA2200	0.179 (FG)	3.5 mm–6.5 mm	Cl	BCC	Ci	SLS	A/E	C, T	S	5 mm/min, 1 mm/min
13	Bernal [255]	2012	F, E	Thiol-ene, Poly-urethane foams	0.1	Unc. (strut to dia. aspect ratio of 8–9)	Cl (2 × 2, appears single layer)	Pyra-like/half-BCC	Ci, Hier. (foam)	Unc.	A/E	C	S	2 × 10^−4^/s
14	Bhat [170]	2023	E	PA12	0.201–0.308	14 mm	Cl (2 × 2 × 2)	TPMS (Schwarz Prim) combined with nested BCC, R-Dod, and octet	Ci	MJF	-	C	S, D	5 mm/min, 50 mm/min, 100 mm/min
15	Bolan [334]	2023	E	Resin (aqua blue, nylon-green tough)	0.02–0.74	Varies (overall Cl 76.2 mm edge)	Cl (3 × 3 × 3–6 × 6 × 6)	Octet	Ci	SLA	-	C	S	0.01/s
16	Bonatti [335]	2019	F, E	SS316L	0.01–0.85	Unc.	Single, Cl (approx. 3 × 3 × 3 and 7 × 7 × 7)	TPMS sheet-like (SC, FCC, BCC)	-	SLM	A/S, A/E	C, H	S	0.0167/s
17	Campanelli [336]	2014	E	Ti6Al4V	0.2234–0.5822	2.0 mm, 2.5 mm, 3.0 mm	Cl (approx. 8 × 8 × 8)	FCC	Sq	SLM	-	C	S	0.5 mm/min
18	Cao [10]	2018	F, E	SS316L	0.06–0.15	10 mm	Cl (3 × 3 × 3)	R-Dod	Ci (dia. varies)	SLM	A/E	C	S	0.9 mm/min
19	Cao [253]	2020	F, E	SS316L	0.12	8 mm	Cl (3 × 3 × 3)	R-Dod	Ci (dia. varies)	SLM	A/E	C	S, D	10^−3^/s, 750/s to 1250/s
20	Cao [254]	2020	F, A	SS316L	0.05–0.2	8 mm	Cl (3 × 3 × 3)	R-Dod	Ci (dia. varies)	SLM	A/E	C	S, D	0.001–3000/s
21	Carassus [110]	2020	F	Ti6Al4V	0.2–0.3	10 mm	Cl (4 × 4 × 4)	TPMS Sheet (Diam, Gyr), TPMS skeletal (Diam, Gyr)	-	SLM	R	C	D	20 m/s
22	Cetin [122]	2019	F, E	AlSi10Mg	Unsp. (strut dia. 1 mm–5 mm)	17.14 mm–40 mm	Cl (1 × 1 × 3, 1 × 1 × 4, 1 × 1 × 5, 1 × 1 × 6, 1 × 1 × 7)	BCC, BCC-Z	Ci	DMLS	Ab	C	S, D	2 mm/min, 10 m/s
23	Chen [337]	2018	F, A	Al7075/AlSi10Mg	0.144–0.199	Unc.	Cl (54 on layer × 9 layers)	NPR (Pyra re-entrant cell variations)	Ci, HC	SLM	LS	C	S	Unc.
24	Choy [197]	2017	E	Ti6Al4V	0.083–0.764	approx. 2 mm	Cl (varies, overall size approx. 11 × 13 × 14 mm^3^)	SC, honeycomb	Ci	SLM	-	C	S	0.05/min
25	Choy [196]	2017	E	Ti6Al4V	0.35–0.62 (FG)	approx. 2 mm	Cl (varies, overall size 14.2 × 13.0 × 11.6 mm^3^)	SC, honeycomb	Ci	SLM	-	C	S	0.05/min
26	Cui [244]	2018	F, E	VeroWhite Plus	Unsp. (thck. 1 mm)	4 mm	Cl (4 × 4 × 4)	SC/conventional open-cell foam	Sq	Unsp.	Ab	C	S	1 mm/min
27	Della Ripa [338]	2021	F, E	Fabbrix (nylon-CF), AlSi10Mg	Unc. (dia. 1.5 mm)	9 mm	Cl (3 × 3 × 3)	Octet, variations in octet, Kelvin	Ci	FDM, SLS	R	C	S	1 mm/min
28	Dong [88]	2015	E	Ti6Al4V	0.02–0.16	strut length 7 mm–25 mm	Cl	Octet	Sq	WJC	-	C	S	3 × 10^−4^/s
29	Doty [339]	2012	E	Photopolymer	0.064–0.273	1.2 mm, 12.5 mm	Cl (Unc.)	Pyra-like/half-BCC (small scale, large scale, hierarchical)	Ci	Coll. UV	-	C	S	8 × 10^−4^/s
30	Duan [156]	2020	F, E	SS316L	0.1–0.3	5–16.2 mm	Single, Cl (5 × 5 × 5)	(Plate-based) Novel design family	-	SLM	A/S, A/E	C	S	0.000444/s
31	Epasto [311]	2019	E	Ti6Al4V ELI	0.09–0.23	2 mm–4 mm	Cl (6 × 6 × 12, 8 × 8 × 16, 6 × 6 × 12)	R-Dod	Ci	EBM	-	C	S, D	1 mm/min, 1–2 m/s
32	Evans [340]	2010	F, E, A	Ni	0.01–0.10	Unsp. (A/L^2^ = 0.0015–0.05)	Unsp. (single layer)	Pyra	HC	Coll. UV and E-De	A/E	C	S, D	0.0007/s, 100 m/s
33	Fan [237]	2021	E	Ti6Al4V	FG (avg 0.30), 0.15–0.45	3 mm	Cl (6 × 6 × 6)	TPMS sheet (Gyr, Neo, Schwarz Prim)	-	SLM	-	T, C	S	2 mm/min (T), 1 mm/min (C)
34	Gültekin [268]	2022	F	6061T6 aluminum	Unc. (strut dia. 1.18 mm)	Unc., appears 10 mm	Cl (unc., single layer)	Pyra, semicircle, cross semicircle, Kagome, 2D honeycomb	Ci, HC	-	R	C	D	3.5 m/s
35	Habib [143]	2018	F, E	PA12	0.15	10 mm	Cl (5 × 5 × 5)	Circ, Octag, strengthened Octag, Kelvin, R-C-Octah, SC	Ci	MJF	A/E	C	S	5 mm/min
36	Habib [144]	2019	F	PA12	0.15 and FG	5 mm	Cl (5 × 6 × 6 (honeycomb: 7 × 6 cluster))	Octag, Kelvin, Honeycomb	Ci	MJF	A/E	C	D	3.5 m/s
37	Hammetter [341]	2013	F, A	Thiol-ene	0.02–0.40	Unc. (L/D = 2.5–20)	Cl (varies)	Pyra, “Diamond”, “Hourglass”	Ci	-	Ab	C	S	Unc.
38	Hao [342]	2019	E	PA12, GFR PA12	Unsp. (dia. 1 mm)	10 mm	Cl (4 × 4 × 4)	Circ	Ci	SLS	-	C	S	Unsp.
39	Harris [343]	2020	F, E	SS316L	0.115–0.305	~6.50 mm	Cl (3 × 2 × 5)	Origami (stacked Miura-ori)	-	SLM	A/S	C	S	0.001/s
40	Jiang [344]	2021	F, E	PA12	0.15–0.30	Unc.	Cl (3 × 3 × 5)	Shell (uniform foam, graded binder foam, graded thickness foam, hybrid graded foam)	-	MJF	A/E	C	S	5 × 10^−4^/s
41	Jin [129]	2019	F, E	Ti6Al4V	0.027–0.476	5 mm	Cl (2 × 2 × 2)	Dfcc, Dhex, octet, BCC	Ci	SLM	LS	C	D	1000/s
42	Jin [100]	2021	F, E	Ti6Al4V	0.119	5 mm	Cl (4 × 4 × 4)	BCC, octet	Ci	SLM	An/LS	C	S	10^−3^/s
43	Kandasamy [345]	2023	E	PA12	0.1, 0.2, 0.3	strut length 5 mm	Cl (dim.: 50 × 50 × 25 mm^3^)	Kelvin	Ci	SLS	-	C	S	0.001/s, 0.013/s, 0.133/s
44	Kaur [78]	2017	F, E	PLA, CFRPLA, Nylon 618	Unsp. (square 1 mm diag.)	5 mm	Cl (7 × 7 × 10 mm^3^)	Octet, Octah	Sq	FDM	An	C	S	2 mm/min (3 × 10^−3^/s)
45	Kohnen [99]	2018	E	SS316L	0.33	2.8 mm	Cl (5 × 5 × 5, 5 × 5 × 14)	FCC-Z, hollow sphere	Ci	SLM	-	C, T, F	S	0.001/s (C), 0.001/s (T), 32 Hz (F)
46	Leary [120]	2016	F, E	AlSi12Mg	0.085-0.206 (const 1 mm dia. strut)	7.5 mm	Cl (10 × 10 × 10)	BCC, BCC-Z, FCC, FCC-Z, FBCC-Z	Ci	SLM	Unc.	C	S	10^−3^/s
47	Leary [121]	2018	F, E	Inconel 625	0.02–0.10	2 mm–4 mm	Cl (10 × 10 × 15)	BCC, BCC-Z, FCC, FCC-Z	Ci	SLM	Unc.	C	S	10^−3^/s
48	Lei [124]	2019	F, E	AlSi10Mg	0.05–0.06	10 mm	Cl (5 × 5 (×1, ×3, ×5, ×7))	BCC, BCC-Z	Ci	SLM	A/E	C	S	0.5 mm/min
49	Li [346]	2019	F, E	AISI 4130	0.10–0.30	4 mm	Cl (3 × 3 × 3, 5 × 5 × 5)	SC-AFCC	Ci	SLM	Au	C	S	0.001/s
50	Li [235]	2021	F, E	SS316L	0.3	4 mm	Cl (5 × 5 × 5)	TPMS (Gyr)	-	SLM	LS	C	S, D	2 × 10^−5^ m/s, 6.85 m/s, 5050 m/s
51	Liang [239]	2021	E	SS316L	0.25–0.40	2.5 mm	Cl (8 × 8 × 8)	TPMS sheet (Prim, Gyr)	-	SLM	-	C	S	0.001/s
52	Ling [133]	2019	F, E	Polymer (“standard grey” resin, “durable” resin)	0.13–0.45	Approx. 14 mm (strut length 10 mm)	Cl (4 × 4 × 4)	Octet	Ci	SLA	Ab	C	S, D	5.7 mm/min (0.0167/s), 3 m/s (53/s)
53	Lv [171]	2020	F, E	PA2200/Nylon 12	0.15–0.20	89.2 mm	Single	Octet	Ci, Hier. (tubular re-entrant)	SLS	A/E	C	S	0.2 mm/min
54	Ma [44]	2020	E	CuCrZr	0.1–0.2	4 mm–6 mm	Cl (4 × 4 × 4)	TPMS (Diam)	-	SLM	-	C	S	2 mm/min
55	Mahbod [158]	2019	F, E, A	Polymer	Unsp. (rad. = 1 mm–1.4 mm)	Unsp. (strut length 3.4–5 mm)	Cl (approx. 3 × 3 × 3)	Novel double Pyra Dod	Ci	SLA	An/LS	C	S	0.5 mm/min
56	Maskery [49]	2016	E	AlSi10Mg	0.22, FG	3 mm	Cl (6 × 6 × 6)	BCC	Ci	SLM	-	C	S	0.03 mm/s
57	Maskery [98]	2017	E	AlSi10Mg	0.22	3 mm–9 mm	Cl (2 × 2 × 2, 3 × 3 × 3, 4 × 4 × 4, 6 × 6 × 6)	TPMS (Double Gyr)	-	SLM	-	C	S	0.009 mm/s
58	McKown [119]	2008	E	SS316L	0.029–0.166	1.5 mm, 2.5 mm	Cl (8 × 8 × (7, 8, 11) or 13 × 13 × (7, 11, 13))	BCC, BCC-Z	Ci	SLM	-	C	S, D	0.5 mm/min (El), 1 mm/min (Plas), D varies (1–500 mm/min, 17.1 m/s–68.9 m/s)
59	Mieszala [48]	2017	E	IP-Dip polymer and NiB coating (E-De)	0.06–0.45	Approx. 5–10 um	Cl (approx. 4 × 4 × 4)	SC, cubic with braces x2, hexagonal truss, shape-optimized honeycomb	Unc., appears Ci	3D DLW	-	C	S	Disp.-controlled at 20 nm/s
60	Miralbes [210]	2022	E	ABS	0.3–0.5	5 mm	Cl (10 × 10 × 10)	TPMS sheet (Gyr, Diam, Lidinoid, Neo, Schwarz Prim, Split P)	-	FFF	-	C	S	5 mm/min
61	Mueller [177]	2018	E, A	RGD525, FLX9695	Rel. Vol.: 0.12	32.28 mm, 60.57 mm	Single, Cl (3 × 3 × 3)	Kelvin	Ci	MJT	-	C	S	10 mm/min
62	Mueller [134]	2019	F	Al-6101-T6	Unc.	Unc.	Cl (5 × 5 × 5 or 8 × 8 × 8)	Voronoi, octet, Delaunay, SC	Ci	-	A/E	C	D	10^−6^/s to 10^4^/s
63	Nasrullah [160]	2020	F	AlSi-12	0.05–0.30	10 mm	Single	Kagome, Tetra, Pyra, octet, SC, Tr-Pyra, Octah, R-C-Octah, R-Dod, open-cell, twisted-octet	Ci	-	LS	C	D	9 m/s
64	Nazir [225]	2021	F, E	PA12	0.1468–0.1491, 0.1625	10 mm, 20 mm	Single	Kelvin, Modified Kelvin (fillets)	Ci	MJF	An	C	S	1–2 mm/min
65	Novak [208]	2021	F, E	SS316L	0.16–0.21	Unc.	Cl (cyl. dia. 20 × 20 mm^2^)	TPMS (Schwarz Diam, Schoen Gyr)	-	PBF	LS	C	S	0.1 mm/s
66	Ozdemir [245]	2016	E	Ti6Al4V	0.137–0.166	5 mm	Cl (1 × 5 × 5 and 5 × 5 × 5)	SC, Diam, Re-entrant cubic	Ci	EBM	-	C	S, D	0.1–0.2 mm/min, 5–21 m/s, 80–250 m/s
67	Ozdemir [241]	2017	F	Ti6Al4V	0.137–0.166	5 mm	Cl (1 × 5 × 5 and 5 × 5 × 5)	Diam, Re-entrant cubic	Ci, Sq	-	LS	C	S, D	0.2 mm/min, 7.6 m/s, 178 m/s
68	Park [240]	2022	E	CoCrMo	0.255–0.281	2.5 mm	Cl (2 × 2 × 2)	TPMS sheet (Neo, IWP)	-	LPBF	-	C	S	1 × 10^−3^/s
69	Schaedler [347]	2011	E	Ni alloy	0.0001–0.007	Unsp. (strut length 1–4 mm)	Cl	BCC	HC	Coll. UV and E-P	-	C	S	10 um/s
70	Shen [123]	2009	E	SS316L	0.05–0.06	2.5 mm	Cl (unc., appears approx. 8 × 8 × 8, 40 × 8 × 8)	BCC, BCC-Z	Ci	SLM	-	B, C	S, D	1 mm/min–3 m/s (C), 0.25 mm/min–4 m/s (B)
71	Shen [348]	2021	E	Zirconia	0.067–0.336	2–5 mm	Cl (2 × 2 × 2–5 × 5 × 5)	TPMS sheet (Prim, Gyr, IWP, S14)	-	DLP	-	C	S	0.02 mm/min
72	Smith [126]	2011	E	SS316L	0.035–0.159	1.25–2.5 mm	Cl (8 × 8 × 8, 10 × 10 × 10, 13 × 13 × 13, 15 × 15 × 15)	BCC, BCC-Z	Ci	SLM	-	C	S, D	0.5 mm/min (El), 1 mm/min (Plat/Dens), 4.1 m/s–32 m/s
73	Smith [125]	2013	F, E	SS316L	0.035–0.16	1.25 mm–2.5 mm	Cl (8 × 8 × 8, 10 × 10 × 10, 13 × 13 × 13, 16 × 16 × 15)	BCC, BCC-Z	Ci	SLM	A/S	C	S	0.5 mm/min (El), 1 mm/min (Plat/Dens)
74	Song [132]	2019	F, E	Photo-sensitive resin	Various (const. 1.11 mm dia. strut)	8.282 mm	Cl (3 × 3 × 3)	Octet, variations in octet	Ci	SLA	An	C	S, O	2 mm/min (3 × 10^−3^/s)
75	Sun [232]	2022	F, E	Ti6Al4V	0.096–0.327 (four thck.: 0.20–0.40 mm)	5 mm	Cl (4 × 4 × 4)	TPMS sheet (Prim, Gyr, Diam)	-	SLM	A/E	C	S	2 mm/min
76	Tallon [223]	2020	F, E	Maraging steel	0.126	5 mm	Cl (4 × 4 × 4)	R-Dod	Ci	L-PBF	LS	C	S	1.3 mm/min
77	Tancogne-Dejean [251]	2016	F, E	SS316L	0.05–0.50	3.08 mm	Cl (7 × 7 × 7)	Octet	Ci (dia. varies)	SLM	Ab	C	S, D	10^−3^/s, 10^3^/s
78	Tancogne-Dejean [252]	2018	F, E	SS316L	0.1–0.3	3 mm	Cl (7 × 7 × 7 or 5 × 5 × 5)	BCC	Ci (dia. varies)	SLM	A/E, A/S	C	S, D	3 mm/min, 10 m/s
79	Viccica [349]	2022	F, E	PA2200/PA12	Fractal design	Fractal design	Cl (fractal design)	3D Greek cross (fractal design)	Ci	SLS	R	C	S, D	5 mm/min (S), 7.5 m/s (D)
80	Wang [155]	2018	E, A	PLA	0.174–0.374	Approx. 7.5 mm	Cl (overall dims 40 × 40 × 60 mm^3^)	(Plate-based) Random cell, Tetrak, hexagonal prism, Rect. prism, clipped Rect. prism	-	FDM	-	C	S	0.001/s
81	Wang [243]	2020	F, E, A	PA12	0.054–0.062	approx. 17 mm	Cl (3 × 3 × 3)	Cross-chiral honeycomb	Sq	SLS	Ab	C	S, D	2 mm/min (0.067/s), up to 30 m/s
82	Wang [238]	2020	F, E	SS304	0.2	Unc. (appears to be 6 mm)	Cl (approx. 1 × 4 in cyl. shell shape with outer dia. 30 mm)	TPMS sheet (Gyr)	-	SLM	A/E	C	S	1.4 mm/min (0.001/s)
83	Wang [215]	2021	F	SS316L		4 mm	Cl (8 × 8 × 5)	TPMS sheet (Schwarz Prim, IWP, Neo)	-	-	A/E	C	D	1 m/s
84	Xiao [329]	2015	F, E	Ti6Al4V	0.129, 0.164	1.5 mm, 2.5 mm	Cl (6 × 6 × 6, 10 × 10 × 10)	R-Dod	Ci	EBM	An/LS	C	S	0.9 mm/min (10^−3^/s)
85	Xiao [102]	2018	F, E	Ti6Al4V	FG (0.139 to 0.224)	3 × 3 × 2 to 3 × 3 × 4 mm^3^	Cl (8 × 8 × 9)	R-Dod	Ci	SLM	LS	C	S, D	10^3^/s, 500/s, 1000/s
86	Xiao [43]	2018	E	SS316L	0.10–0.30	2.25 mm	Cl (8 × 8 × 8)	ECC, FCC, VC	-	SLM	Ab	C	S	1 mm/min
87	Yang [204]	2019	F, E	SS316L	0.15, FG	4 mm	Cl (4 × 4 × 4)	TPMS (Gyr)	-	SLM	De	C	S	0.02 mm/s (0.001/s)
88	Yang [233]	2019	F, E	Ti6Al4V	approx. 0.04–0.3	8 mm–20 mm	Cl (2 × 2 × 2–5 × 5 × 5)	TPMS (Gyr)	-	SLM	-	C	S	4 mm/min
89	Yang [159]	2020	F	HSSG350, AA6063, Ti6Al4V	0.160–0.341	7.5 mm	Cl (3 × 3 × 3)	CLL, octet, BCC-6H	Ci	-	LS	C	D	1 m/s, 10 m/s
90	Yang [236]	2022	F, E	SS316L	0.05–0.20 (FG)	4 mm	Cl (5 × 5 × 5)	TPMS (Gyr)	-	LPBF	A/E	C	S	0.02 mm/s
91	Yazdani Sarvestani [157]	2018	F, E, A	PLA	0.30–0.50	1.8 mm, 3 mm	Cl (7 × 1 × 1, 7 × 2 × 2, 5 × 5 × 2)	Plate-based (octet, SC, isomax (octet + SC))	-	FDM	Au	B, C	S, D	0.5 mm/min (S, B), 10 kN (22 kg impactor, D)
92	Yin [112]	2020	F, E	CX stainless steel	Unsp. (thck. 0.15 mm–0.75 mm)	4 mm	Cl (4 × 4 × 4–7 × 7 × 7)	TPMS sheet (Prim, FRD, IWP, Gyr)	-	DMLS	LS	C	S, D	2 mm/min, 5–50 m/s
93	Yu [172]	2022	F, E	Tough2000 resin	0.1657–0.2071	8 mm	Cl (4 × 4 × 4)	R-Dod, octet	Ci	SLA	Ab	C	S	10^−3^/s
94	Yuan [350]	2019	E	CNT/PA12	0.09–0.30	Unc.	Cl (5 × 5 × 6)	BCC-6H, BCC-12H	-	SLS	-	C	S	6 mm/min
95	Zhang [111]	2018	F, E	SS316L	0.11–0.39	2.5 mm (BCC), 4 mm (TPMS)	Cl (6 × 6 × 6 (BCC), 5 × 5 × 5 (TPMS))	TPMS sheet (Prim, Gyr, Diam), BCC	Ci	SLM	A/E	C	S	10^−3^/s
96	Zhang [205]	2020	F	SS316L	FG (0.05–1)	3 mm	Cl (2 × 2 × 2, 5 × 5 × 5)	TPMS (IWP, Prim)	-	SLM	De	C	S	1 mm/min
97	Zhang [351]	2021	F, E, A	AlSi10Mg	Unc. (four dia.: 1.2 mm, 1.6 mm, 1.9 mm, 2.2 mm)	10 mm	Cl (5 × 5 × 5)	Var. on BCC with decr./incr. in number of struts, adding Z struts, adding partial FCC struts	Ci	SLM	Ab	C	S	2 mm/min
98	Zhao [101]	2018	E	Ti6Al4V	0.10–0.30	4 mm	Cl (4 × 4 × 4)	BCC, TPMS (BCC)	Ci	SLM	-	C	S	1 mm/min
99	Zhao [114]	2020	F, E	Ti6Al4V	FG (avg 0.20)	4 mm	Cl (5 × 5 × 5)	TPMS sheet (Prim, Gyr)	-	SLM	A/S	C	S	2 mm/min
100	Zhong [50]	2019	F, E	SS316L	0.125–0.404	5 mm	Cl (5 × 5 × 5)	BCC, Diam, Unique Tetrak	Ci	SLM	Ab	C, T	S	6 mm/min (C and T)

Unc./Unsp.—Unclear/unspecified in paper; avg—average; cyl.—cylinder/cylindrical; dia.—diameter; dim.—dimensions; Rel.—relative; Thck.—thickness; Var.—varies/various/variations; and Vol.—volume. **Methods**: F—FEA, E—experiment, A—analysis. Material: CFRPLA—Carbon Fiber Reinforced PLA, ELI—Extra Low Interstitial, GFR—Glass Fiber Reinforced, HDPE—High Density Polyethylene, MWCNT—Multi-Walled Carbon Nanotube, PPR—Polypropylene Random Copolymer. **Relative Density**: FG—functionally graded. **Single/Cluster**: Cl—cluster. **Topology**: Circ—circular, Diam—diamond, Dod—dodecahedron, Gyr—gyroid, Neo—neovius, NPR—Negative Poisson’s Ratio, Octah—octahedron, Octag—octagonal, Ori—origami, Prim—primitive, Pyra—pyramid/pyramidal, Rect.—rectangular, R-Dod—rhombic dodecahedron, R-C-Octah—rhombicuboctahedron, SC—simple cubic (cubic), Tetra—tetrahedron, Tetrak—tetrakaidecahedron, Tr-C-Octah—truncated cuboctahedron, Tr-Pyra—truncated pyramid, Tr-SC—truncated simple cubic. **Cross-section**: Ci—circle, HC—hollow circle, Sq—square, Hier.—hierarchical. **Manuf. Process**: SLM—Selective Laser Melting, EBM—Electron Beam Melting, SLS—Selective Laser Sintering, SLA—Stereolithography, FDM—Fused Deposition Modeling, FFF—Fused Filament Fabrication, MJF—Multi-Jet Fusion, WJC—Water Jet Cutting, Coll. UV—Collimated UV, 3D DLW—3D Direct Laser Writing, L-PBF—Laser Powder Bed Fusion, DMLS—Direct Metal Laser Sintering, E-P—Electroless-Plating, E-De—Electro-Deposition. **Software**: LS—LS-DYNA, A/E—ABAQUS/Explicit, A/S—ABAQUS/Standard, Ab—ABAQUS, An—Ansys, Au—AUTODYN, R—RADIOSS, De—DEFORM. **Load**: C—compression, T—tension, H—hydrostatic, F—fatigue, B—bending. **Numerical Strain Rate/Speed**: El—elastic, Plat—plateau, Plas—plastic, Dens—densification. **Rate**: S—static, D—dynamic, O—other.
